# Identification of a Core miRNA-Pathway Regulatory Network in Glioma by Therapeutically Targeting miR-181d, miR-21, miR-23b, β-Catenin, CBP, and STAT3

**DOI:** 10.1371/journal.pone.0101903

**Published:** 2014-07-09

**Authors:** Ronghong Li, Xiang Li, Shangwei Ning, Jingrun Ye, Lei Han, Chunsheng Kang, Xia Li

**Affiliations:** 1 College of Bioinformatics Science and Technology, Harbin Medical University, Harbin, China; 2 Department of Neurosurgery, Tianjin Medical University General Hospital, Laboratory of Neuro-Oncology, Tianjin Neurological Institute, Laboratory of Neurotrauma, Variation and Regeneration, Ministry of Education and Tianjin Municipal Government, Tianjin, China; Beijing Tiantan Hospital, Capital Medical University, China

## Abstract

The application of microRNAs (miRNAs) in the therapeutics of glioma and other human diseases is an area of intense interest. However, it’s still a great challenge to interpret the functional consequences of using miRNAs in glioma therapy. Here, we examined paired deep sequencing expression profiles of miRNAs and mRNAs from human glioma cell lines after manipulating the levels of miRNAs miR-181d, -21, and -23b, as well as transcriptional regulators β-catenin, CBP, and STAT3. An integrated approach was used to identify functional miRNA-pathway regulatory networks (MPRNs) responding to each manipulation. MiRNAs were identified to regulate glioma related biological pathways collaboratively after manipulating the level of either post-transcriptional or transcriptional regulators, and functional synergy and crosstalk was observed between different MPRNs. MPRNs responsive to multiple interventions were found to occupy central positions in the comprehensive MPRN (cMPRN) generated by integrating all the six MPRNs. Finally, we identified a core module comprising 14 miRNAs and five pathways that could predict the survival of glioma patients and represent potential targets for glioma therapy. Our results provided novel insight into miRNA regulatory mechanisms implicated in therapeutic interventions and could offer more inspiration to miRNA-based glioma therapy.

## Introduction

Glioma is among the most common and aggressive forms of primary brain cancer in adults, characterized by rapid and invasive growth and poor prognosis [1]. Advances in glioma treatment over the past two decades have resulted in only a modest improvement in survival rate [2], and there is therefore an urgent need to develop novel treatments such as gene therapy that do not depend on conventional pharmacological approaches.

MicroRNAs (miRNAs) are endogenous, small, non-coding RNAs that regulate gene expression at the post-transcriptional level [3]. Many studies have demonstrated the role of miRNAs in a variety of human diseases including glioma [4], [5], and the clinical potential of miRNAs as therapeutic agents and targets has elicited considerable interest [6], [7]. For example, overexpression of miR-181d has been shown to inhibit the growth of glioma cells [8], [9], while miR-21 or -23b knockdown suppressed glioma invasion and improved prognosis [10], [11]. However, a significant barrier for the clinical application of miRNAs is the limited knowledge of the consequences of targeting individual miRNA.

Several recent studies have found that modulating a specific miRNA can not only alter the expression level of target mRNAs, but can also cause global alterations in levels of mRNAs targeted by other miRNAs [12], implying the existence of miRNA networks in which individual miRNAs can have both direct and indirect targets and act cooperatively to regulate gene expression [13]. For instance, simultaneously inhibiting miRNA-10b and -21, both of which are expressed at elevated levels in glioma, more effectively suppressed human glioma cell proliferation and invasion than the inhibition of either miRNA alone [14]. It has also been reported that modulating the activity of specific transcription factors (TFs) can inhibit tumorigenesis by altering the expression of endogenous miRNAs [15]. Specifically, suppression of β-catenin, signal transducer and activator of transcription3 (STAT3), and CREB-binding protein (CBP) has been shown to prevent the growth and metastasis of glioma [16]–[18], though few studies have examined changes in the expression of miRNAs resulting from these TF-targeted therapeutic interventions. Since a single miRNA can have multiple targets, identifying the affected pathways is useful for interpreting the function of miRNAs in relation to a specific biological process [19]. However, it’s still a great challenge to identify miRNA-pathway regulatory network responding to therapeutic interventions in glioma, partially due to the lack of paired expression profiles of miRNAs and mRNAs.

In this study, miRNAs and mRNAs were simultaneously profiled using high-throughput sequencing in human glioma cell lines after interfering with the expression of miR-181d, -21, and -23b, as well as β-catenin, CBP, and STAT3. MiRNAs and mRNAs altered by these treatments were used to construct miRNA-pathway regulatory network (MPRN) for glioma. Manipulating the levels of miRNAs and TFs induced global changes in miRNA and mRNA expression, exerting great influence on biological pathways specifically implicated in glioma. Moreover, we identified a core module that was consistently activated by various treatments and could also predict glioma patient survival. These findings provide insight into the role of miRNAs in gliomagenesis and can facilitate the identification of novel therapeutic targets, as well as the development of more effective glioma treatment strategies.

## Materials and Methods

### Reagents, cell culture, and transfections

MiR-181d mimics, and miR-23b and -21 inhibitors as well as control miRNAs, were purchased from Qiagen (Hilden, Germany). Inhibitors for STAT3 (WP1066), CBP (ICG001), and β-catenin (FH535) were from Calbiochem (Darmstadt, Germany); in these experiments, cells were treated with DMSO as a control. The human glioblastoma cell line U87 was obtained from the Academia Sinica cell repository (Shanghai, China). Cells were maintained in Dulbecco’s Modified Eagle’s Medium (Invitrogen, Carlsbad, CA, USA) supplemented with 10% fetal bovine serum and incubated at 37°C and 5% CO_2_. MiRNA and TF transfections were performed using Hiperfect transfection reagent (Qiagen) and Lipofectamine 2000 (Invitrogen), respectively, according to the manufacturer’s recommendations, and cells were harvest 72 h later.

### Target gene identification

Predicted miRNA targets were determined using DIANA-microT (version 3.0) [20], mirSVR [21], PicTar (five-way) [22], RNA22 [23], RNAhybrid [24], TargetScan (version 6.0) [25], PITA (version 6) [26], MirTarget2 (version 4.0) [27], TargetMiner [28], and miRanda [29], and highly efficient miRNA-target interactions that occur in at least four of the ten sources were used for subsequent analyses. A total of 365,539 interactions between 687 miRNAs and 16,212 genes were obtained. β-catenin, CBP, and STAT3 target genes were obtained from available results of Chipseq experiments [30]–[32].

### Patient survival analysis

Paired miRNA and mRNA expression profiles (level 3) and corresponding clinical information from 61 glioblastoma multiforme (GBM) patients that had received adjuvant therapy were downloaded from The Cancer Genome Atlas (TCGA) [33]. Expression profiles were processed with quantile normalization [34], and the average expression of 534 miRNAs and 17,814 mRNAs was obtained from replicate samples for each individual.

### Construction and sequencing of the mRNA library

RNA libraries for mRNA sequencing were prepared with the Illumina standard kit based on the manufacturer’s protocol. Briefly, poly(A) mRNA was isolated from total RNA samples using magnetic oligo(dT) beads and subjected to fragmentation. First strand cDNA was synthesized using random hexamers, followed by double-stranded cDNA synthesis in a reaction containing buffer solution, dNTPs, RNase H, and DNA polymerase I. The cDNA was purified using the QiaQuick PCR purification kit (Qiagen) and resuspended in elution buffer, and after end repair and addition of a single A base, cDNA fragments were ligated to sequencing adapter oligos (Illumina Inc., San Diego, CA, USA). Size selection was performed by agarose gel electrophoresis, generating cDNA libraries ranging from 200–250 bp. After PCR amplification, the mRNA library was sequenced using HiSeq 2000 (Illumina Inc.).

### Estimation of mRNA abundance and identification of differentially expressed mRNAs

Raw mRNA reads were mapped to the human reference genome (hg19) using the TopHat v.1.3.3 program with default settings. The Cufflinks v.1.1.0 program was then used to assemble the reads into transcripts based on gene annotations from Gencode v.9 and estimate their abundance. The expression level of each gene was measured in fragments per kilobase of exon model per million mapped reads (FPKM). In cases where a gene was expressed only in the case or control sample, 0.0001 was added to its expression to compute the fold change. For genes whose expression was equal to or greater than 1 FPKM in both the case and control samples, their differentially expressed fold change threshold was set to 2 in either direction. In addition, genes with expression less than 1 FPKM in one sample but greater than the median expression of all genes in the paired sample were also considered deregulated as a result of the corresponding experimental manipulation.

### Construction and sequencing of the miRNA library

MiRNA libraries were prepared using a method similar to mRNA library construction. Small RNAs ranging from 18 to 30 nucleotides were isolated from total RNA samples by size fractionation and ligated to adapter oligos. After PCR amplification, the library was sequenced using HiSeq 2000 (Illumina Inc.).

### Estimation of miRNA abundance and identification of differentially expressed miRNAs

Raw miRNA sequencing reads were mapped to the reference human genome (hg19) using Bowtie v.0.12.7 with no mismatches permitted. The resulting SAM file was converted to a BAM file with SAMtools v.0.12.7, then transformed into a BED file using bamToBed, from which the depth of each miRNA was derived using the coverageBed tool based on the human miRNA annotation from miRBase v.17. The expression of each miRNA was measured in reads per million reads (RPM). In cases where a miRNA was expressed only in either the case or control sample, 0.0001 was added to its expression to compute the fold change. For miRNAs whose expression was equal to or greater than 1 RPM in both case and control samples, the fold change threshold was set to 2 in either direction. In addition, miRNAs whose expression was less than 1 RPM in one sample but greater than the median for all expressed miRNAs in the paired sample were also considered deregulated by the corresponding experimental manipulation.

### Pathway enrichment analysis

Biological pathways significantly affected by mRNAs that were differentially expressed upon each intervention were identified using the hypergeometric distribution test. The p value for each pathway represented the statistical significance of the overlap between the differentially expressed mRNAs and the annotated genes in the pathway and was calculated as follows:
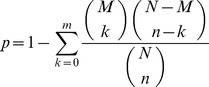
where N represents the total number of annotated genes in all pathways from KEGG PATHWAY database [35], n is the number of differentially expressed mRNAs, M is the number of annotated genes in a specific pathway, and m is the number of differentially expressed mRNAs annotated in a specific pathway. Pathways with p<0.01 were considered significantly influenced by a given intervention. In addition, pathways enriched with the target genes of each differentially expressed miRNA were identified, where N represents the total number of annotated genes in all pathways, n is the number of miRNA target genes, M is the number of annotated genes in a specific pathway, and m is the number of annotated miRNA target genes in a specific pathway. Pathways with p<0.01 were considered candidate target pathways that could potentially be regulated by the differentially expressed miRNA.

### Identification of functional miRNA-pathway regulation

For each therapeutic intervention, pathways significantly influenced by differentially expressed mRNAs and candidate target pathways regulated by each differentially expressed miRNA were determined by pathway enrichment analysis. For each differentially expressed miRNA, candidate target pathways that were also significantly influenced by differentially expressed mRNAs were considered as biologically reliable target pathways. All differentially expressed miRNAs and their reliable target pathways were defined as functional miRNA-pathway regulatory network (MPRN) associated with a particular intervention.

## Results

### Altering the levels of specific miRNAs and TFs causes global changes in miRNA and mRNA expression

Firstly, we investigated the levels of three targeted miRNAs in each manipulation, and found that all the three miRNAs was differentially expressed in accordance with the manipulation, for example, miR-181d was significantly upregulated under +miR-181d (Fig. S1). Therefore, we inferred that the expression amount of miR-181d and other miRNAs could recapitulate normal physiologic levels. Since Dicer and Drosha are two important regulators of miRNA expression [36], we investigated whether the amount of Dicer and Drosha changed global levels of miRNA expression. Firstly, we checked the change in expression of their coding genes, DICER1 and DROSHA, after each manipulation. The result revealed that neither of them was significantly altered, implying that there was no significant difference in the amount of Dicer and Drosha after each manipulation. In addition, we have analyzed the distribution of miRNA expression levels in each sample (Fig. S2A), and observed that there was no significant difference between paired case and control samples of each manipulation(p>0.10; Wilcoxon test), indicating that global miRNA expressions were not significantly influenced by the saturation of Dicer and Drosha. Therefore, though miRNA expressions are regulated by Dicer and Drosha, their influence on the expression alteration of miRNAs in our experiment is not significant. Moreover, we also assessed the distribution of mRNA expression levels in each sample (Fig. S2B), and found that no significant difference existed between paired case and control samples (p>0.10; Wilcoxon test). Therefore, the expression of miRNAs and mRNAs detected in our experiment were reliable to analyze the influence of each manipulation.

**Figure 1:**
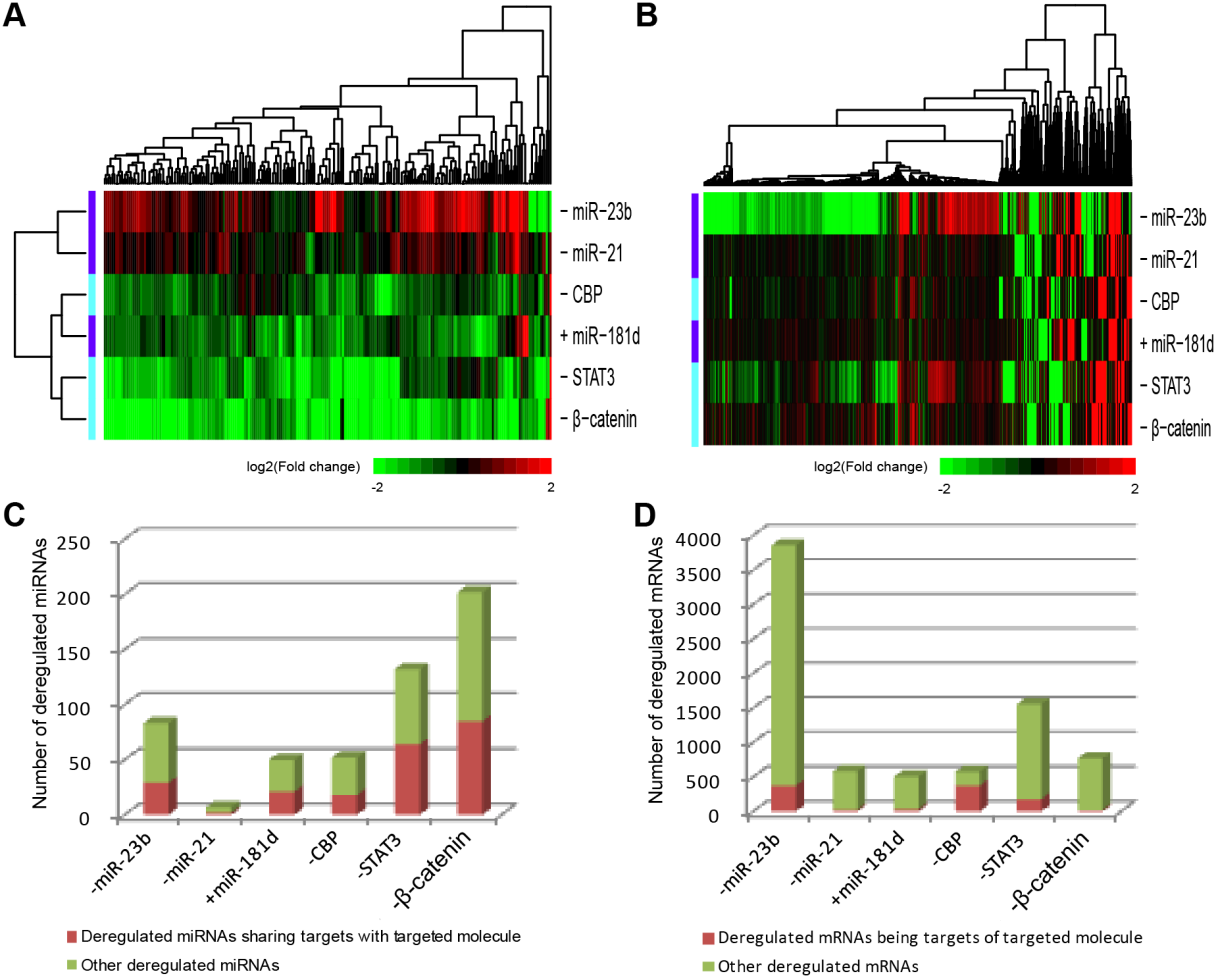
Changes in the expression of significantly deregulated miRNAs and mRNAs after each manipulation. (A) Two-way hierarchical clustering of deregulated miRNAs resulting from all the six experimental manipulations, which are globally sorted into two groups, based on log2 fold changes. (B) One-way hierarchical clustering of deregulated mRNAs resulting from each manipulation based on log2 fold changes. Up- and downregulation of gene expression are represented by red and green colors, respectively, while black indicates no change relative to baseline levels. The number of (C) miRNAs and (D) mRNAs significantly deregulated by each manipulation, with the fraction of these that share targets with the manipulated molecule marked with red.

An examination of the fold change of miRNAs and mRNAs expressed in at least one case or control sample of each intervention showed that manipulations targeting both miRNAs and TFs could alter miRNA and mRNA expression (Fig. S2C, D). MiRNAs that were significantly altered by a given condition were hierarchically clustered based on the log2 fold change value (Figure 1A). Notably, the expression of most miRNAs was reduced by the upregulation of miR-181d (+miR-181d) or inhibition of β-catenin (−β-catenin), CBP (−CBP), or STAT3 (−STAT3), while expression levels were increased by downregulation of miR-21 (−miR-21) and -23b (−miR-23b). However, the directionality of the changes in mRNA expression was not always evident (Figure 1B). Moreover, many differentially expressed miRNAs shared target genes with the molecule targeted by a given manipulation (Figure 1C), while most of differentially expressed mRNAs were not direct targets (Figure 1D). Therefore, we inferred that miRNAs are more influenced than mRNAs by the targeted manipulations, and are likely to be important regulators of gene expressions.

### Altering miRNA expression levels causes changes in target mRNA expression

**Figure 2:**
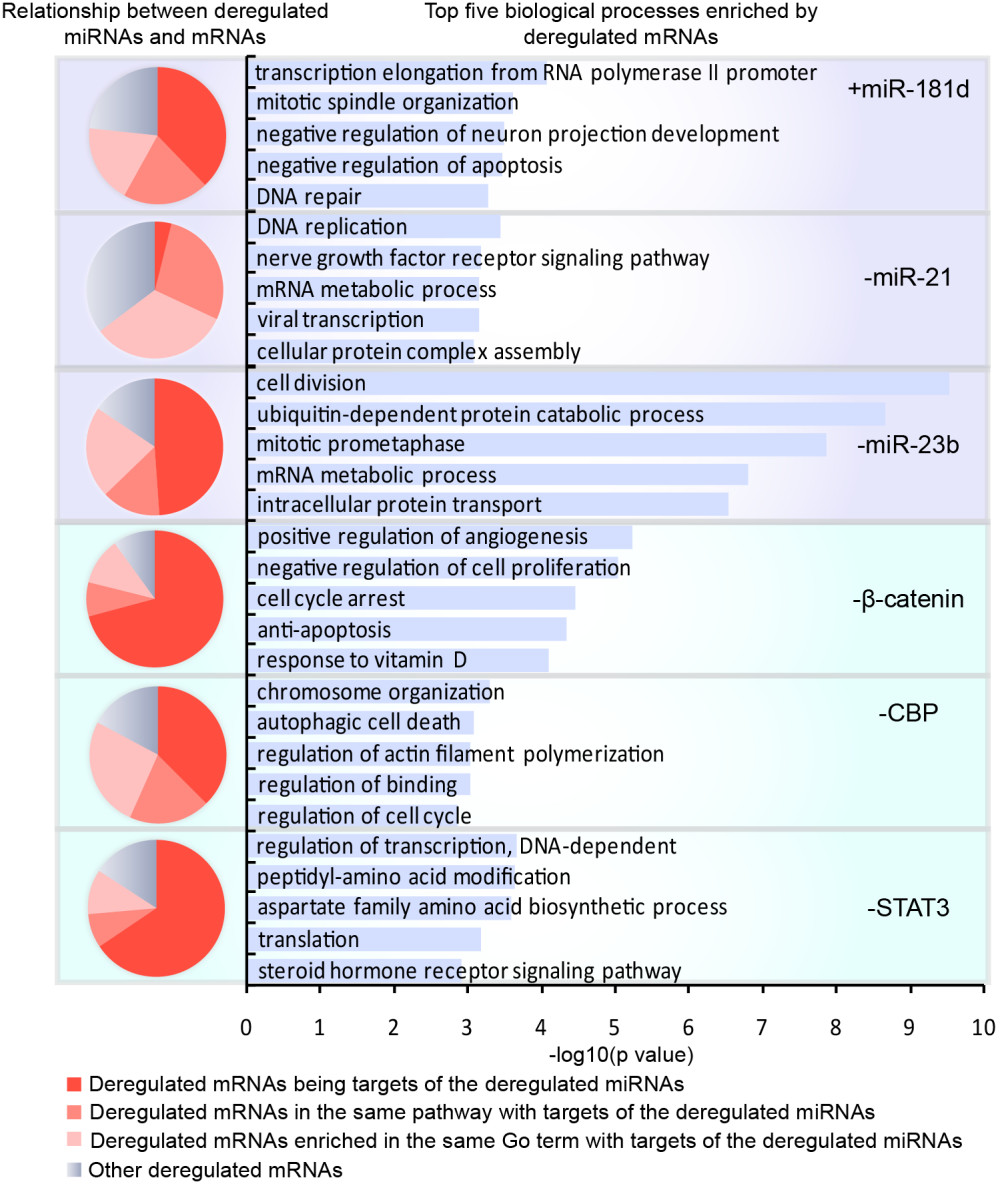
The function of mRNAs with altered expression and their relationship to deregulated miRNAs after each manipulation. The proportion of differentially expressed mRNAs in each type of relationship to deregulated miRNAs is shown in a pie chart, in which the intensity of the red color indicates the degree of association, with a more intense color representing a direct association; blue is used to indicate an unspecified relationship. The five biological processes most significantly enriched by altered mRNAs resulting from each manipulation are listed.

Although most of the differentially expressed mRNAs were not direct targets of the miRNAs or TFs manipulated in this study, a large proportion of them (44.2% on average) were direct targets of miRNAs that had altered expression as a result of a given treatment, especially for the inhibition of β-catenin and STAT3, more than 65% of whose differentially expressed mRNAs were targeted by the deregulated miRNAs (Figure 2). The expression of genes involved in the same biological process are frequently correlated [37]. Therefore, we further investigated the fraction of altered mRNAs in the same KEGG pathway or enriched in the same Gene Ontology (GO) term with direct targets of deregulated miRNAs. On average, 16.2% of altered mRNAs associated with each intervention were in the same pathway with direct targets of the deregulated miRNAs. Biological processes enriched by altered mRNAs in a given manipulation were identified using the Gene Ontology program GOSim [38]. Notably, GO biological processes most significantly affected by differentially expressed mRNAs of these manipulations were specific hallmarks of cancer, including DNA repair (*i.e.*, genomic instability and mutation) for +miR-181d, ubiquitin-dependent protein catabolic process (*i.e.*, dysregulation of cellular energetics) for −miR-23b, DNA replication (*i.e.*, unlimited potential for self-renewal) for −miR-21, angiogenesis for −β-catenin, autophagic cell death (*i.e.*, resisting cell death) for −CBP, and the steroid hormone receptor signaling pathway (*i.e.*, self-sufficiency in growth signals) for −STAT3[39]. This is consistent with the therapeutic effect of all the six manipulations on glioma suppression. Taking into account that nearly 20.2% of the enriched GO terms contained direct targets of deregulated miRNAs, more than 80% of the differentially expressed mRNAs were associated with deregulated miRNAs for all the six manipulations, suggesting that regulation of differentially expressed miRNAs was largely responsible for the altered mRNAs.

### Glioma-specific pathways are targeted by deregulated miRNAs

**Figure 3:**
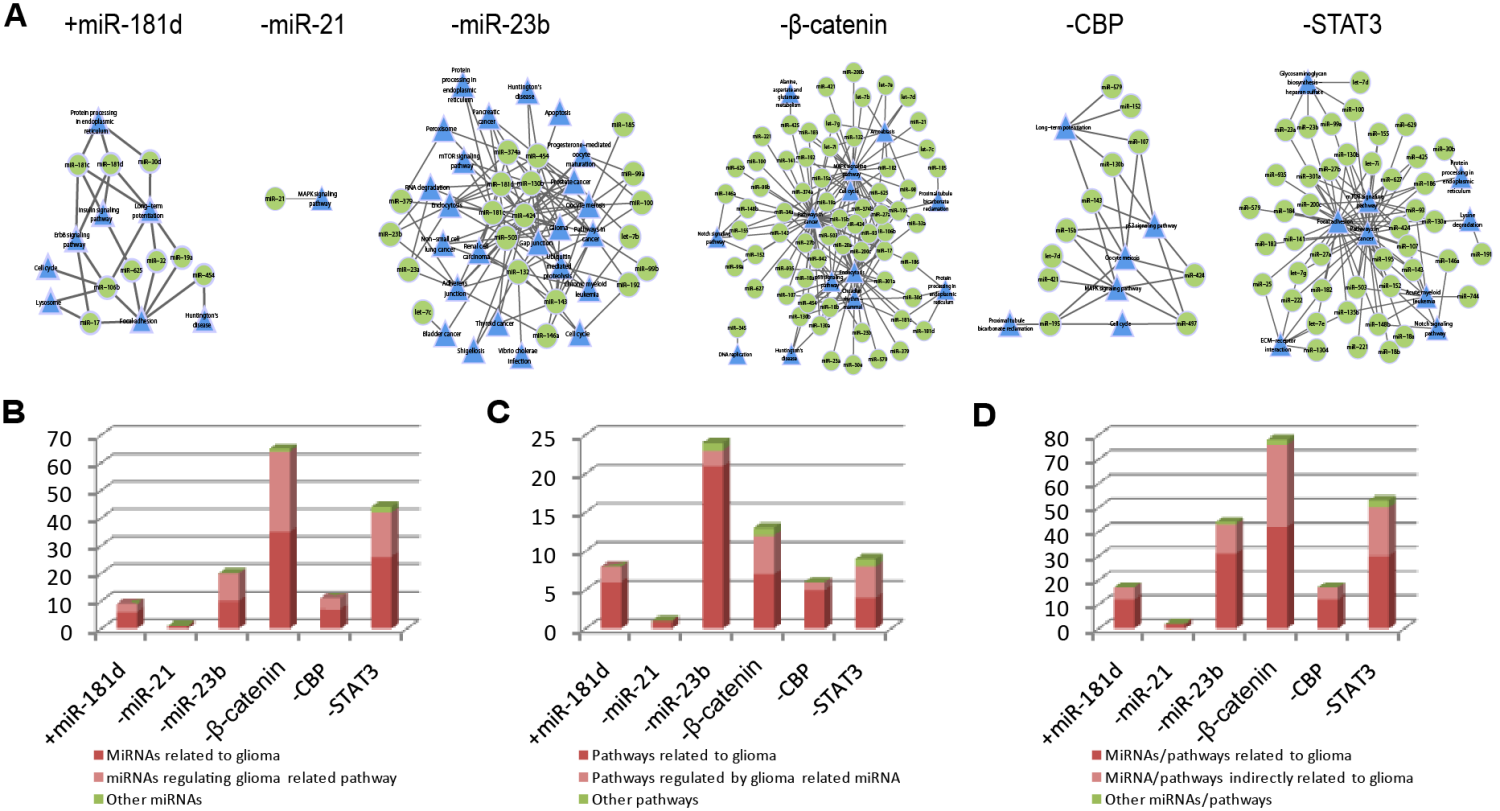
Global views of functional miRNA-pathway regulation networks (MPRNs). (A) Functional MRPNs resulting from each experimental manipulation. Green circles and blue triangles represent functional miRNAs and targeted pathways, respectively. The number and proportion of (B) miRNAs related to glioma and (C) pathways related to glioma in each MRPN, and (D) the overall proportion of miRNAs and pathways related to glioma in each MPRN are shown.

MPRNs were constructed based on differentially expressed miRNAs and mRNAs after targeted manipulating the levels of miR-181d, - 23b, and -21, as well as STAT3, CBP, and β-catenin (Figure 3A). All but –miR21 had networks with more than one regulation of miRNA to pathways, revealing a functional synergism of endogenous miRNAs. For example, the MPRN for +miR-181d contained a total of nine miRNAs and eight distinct pathways, including miRNAs that regulate cell cycle (hsa04110) and focal adhesion (hsa04510). Notably, although glioma pathway (hsa05214) was significantly affected by knockdown of miR-23b, it was not directly regulated by miR-23b, but by seven other miRNAs, i.e., miR-130b, -143, -181c, -181d, -424, -454, and -503, while miR-23b was directly implicated in the regulation of adherens junction (hsa04520), RNA degradation (hsa03018), and endocytosis (hsa04144). Only one functional regulation–the regulation of miR-21 to MAPK signaling pathway–was identified after knocking down miR-21 (−miR-21). After investigating the distribution of differentially expressed mRNAs within this pathway under –miR-21, we found that most of the downregulated genes distributed in the upstream of this pathway, implying the reduced activity of MAPK signaling (Fig. S3A). Since MAPK signaling pathway includes some well known glioblastoma pathogenic genes, such as EGFR [40] *and* PDGFR [41], changes in signal transduction of this pathway could have an important impact on gliomagenesis. In addition, a significant downregulation of HRAS, but no change in the Spry2 transcript level was observed for MAPK signaling pathway, in accordance with a previous report that miR-21 suppresses Spry2 expression at the protein level and thereby disrupts the negative feedback circuit of Ras/MAPK signaling in glioma [42].

An analysis of MPRNs associated with TF targeted manipulations indicated not only the cooperation of differentially expressed miRNAs in regulating various biological pathways, but also the correlation of miRNA expressions and TF activity. For example, the expressions of miR-181c and -181d were decreased after inhibiting β-catenin activity, confirming the positive correlation of miR-181 family members’ expression and β-catenin [43]. MiR-143 and -195 were identified to regulated the largest number of pathways in MPRN activited by CBP inhibition, including cell cycle (hsa04110) and p53 signaling pathway(hsa04115). Since miR-143 and -195 have tumor-suppressor roles in human glioma [44], [45], we inferred that their reduced expression could impair the potential therapeutic effects of CBP inhibition in glioma. Additionally, in MPRN activated by −STAT3, pathways in cancer (hsa05200) were found to be regulated by the largest number of deregulated miRNAs, with effects on angiogenesis, apoptosis evasion, and proliferation. The expression of all genes in this pathway governing angiogenesis and apoptosis were downregulated, including p300/CBP, Glut1, mTOR, and JNK, but upstream genes regulating proliferation such as CyclinD1 and CDK4 were upregulated (Fig. S4). While blocking STAT3 signaling can induce apoptosis and inhibit angiogenesis in tumor cells [46], low levels of STAT3 can still stimulate proliferation by inducing the expression of CyclinD1 and CDK4[47]. In the present study, CyclinD1 was targeted by six deregulated miRNAs that regulated pathways in cancer and were reduced in expression–that is, let-7g and -7i, and miR-195, -424, -503, and -93–suggesting that this could underlie the increased levels of CyclinD1.

Furthermore, we investigated the reliability of the identified functional miRNA-pathway regulations with bootstrap method. 1000 groups of miRNAs and mRNAs were randomly chose as the differentially expressed ones from all the expressed miRNAs and mRNAs for each molecular intervention, with the number of miRNAs and mRNAs in each group equal to that of the real condition. We calculated the identified frequency of each functional miRNA-pathway regulation in the 1000 times randomizations, and found that the average significance of each MPRN was lower than 0.01 (Fig. S5). Therefore, the identified functional miRNA-pathway regulations are representative of each targeted intervention.

In addition, 134 miRNAs were identified from miR2Disease [48], the Human MicroRNA Disease Database [49], and miREnvironment [50] that were previously shown to be associated with glioma, along with 81 pathways that have at least one gene overlapping with glioma pathway from KEGG. After calculating the fraction of miRNAs and pathways being related to glioma in each MPRN, we found that most of the MPRNs activated by targeted manipulations were associated with glioma (Figure 3B–D).

### Functional synergy and crosstalk of MPRNs

**Figure 4:**
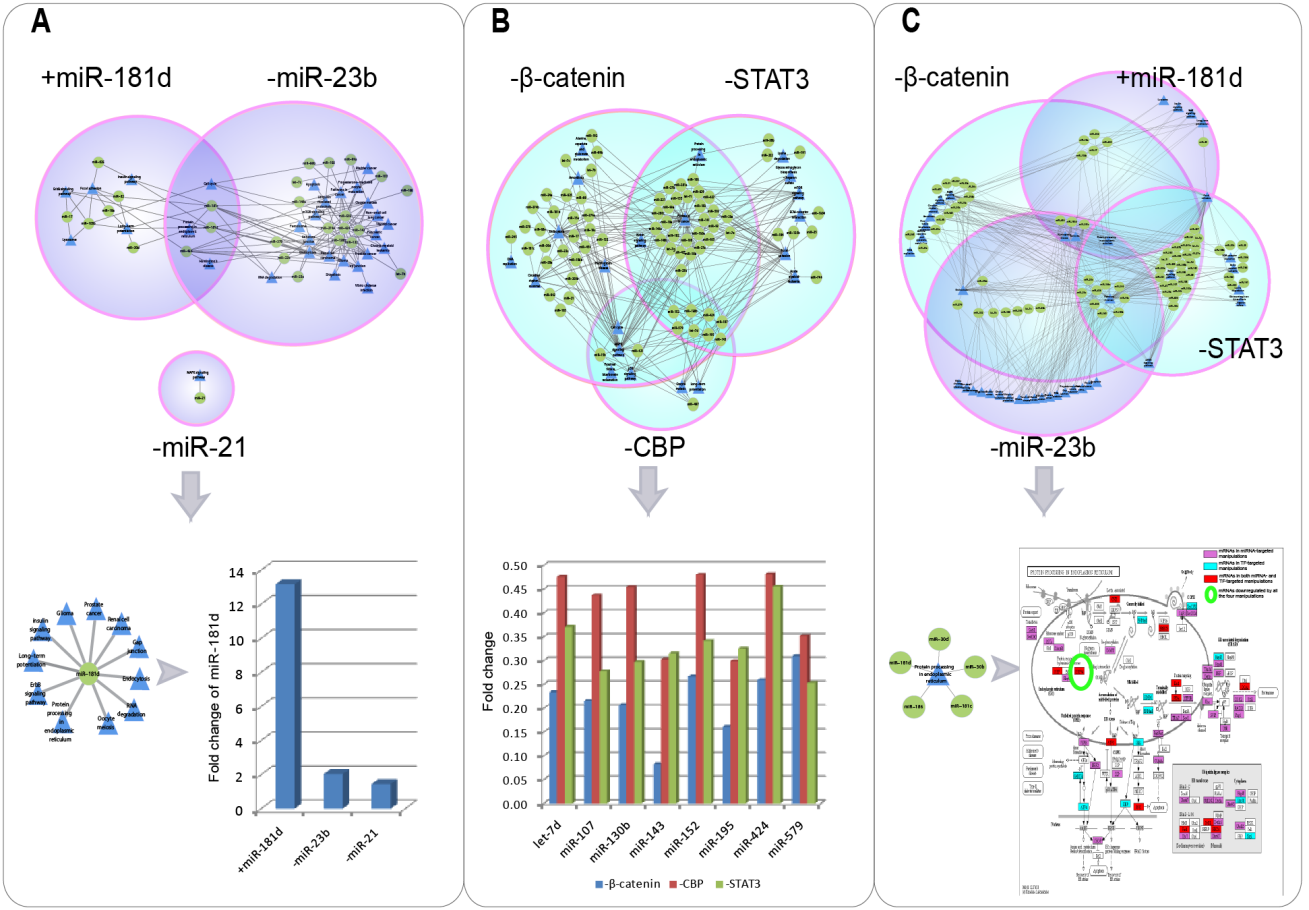
Functional synergy and crosstalk between MPRNs activated by different targeted manipulations. (A) Functional synergy and crosstalk between MPRNs activated by three miRNA targeted manipulations. Since miR-181d was upregulated by all the three manipulations, its target pathways and fold changes are provided. Green circles and blue triangles represent functional miRNAs and targeted pathways, respectively. (B) Functional synergy and crosstalk between MPRNs activated by three TF targeted manipulations. Since eight miRNAs were shared by all the three manipulations, their fold changes under all three manipulations are shown. (C) Example of functional synergy between MPRNs associated with manipulations targeting miRNAs and TFs. Since protein processing in the endoplasmic reticulum pathway was shared by +miR-181d, −miR-23b, −β-catenin, and −STAT3, miRNAs regulating this pathway are shown. The distribution of mRNAs with altered expression in this pathway is shown in the diagram, where purple, cyan, and red rectangles represent mRNAs deregulated by manipulations targeting miRNA, TFs, or both, respectively. HSP90B1 (circled in green) was downregulated by all the four experimental conditions.

Since most of the targeted manipulations activated more than one functional miRNA-pathway regulation, we investigated the functional synergy and crosstalk between MPRNs responding to each manipulation. Excluding −miR-21, which only significantly stimulated the regulation of miR-21 to MAPK signaling pathway, functional synergy was observed between MPRNs activated by miRNA targeted manipulation (+miR-181d and −miR-23b), including three miRNAs, pathways, and regulatory associations in common (Figure 4A). In particular, miR-181d was highly upregulated by all the three miRNA targeted interventions, participating in the regulation of 11 biological pathways. For instance, protein processing in endoplasmic reticulum (hsa04141) was regulated by miR-181d in both +miR-181d and −miR-23b. Moreover, four genes in this pathway were differentially expressed in the same direction under +miR-181d and −miR23b, including upregulated *DNAJB11*, *CKAP4*, and *HSP90B1*, and downregulated *NSFL1C*.

Functional synergy and crosstalk was also observed for MPRNs responding to TF targeted manipulations (Figure 4B). Eight miRNAs (let-7d, and miR-579, -107, -195, -152, -424, -130b, and -143) were shared by the three MPRNs and simultaneously downregulated under all the three manipulations. All of these eight miRNAs but miR-579 and -424 have been reported to be associated with glioma. Moreover, these miRNAs participated in regulating most of the pathways within each MPRN, *i.e.*, all six pathways for −CBP, 10 of 13 pathways for −β-catenin, and six of nine pathways for −STAT3. On the other hand, three pathways were significantly influenced by both −β-catenin and −STAT3, including protein processing in endoplasmic reticulum (hsa04141), pathways in cancer (hsa05200), and Notch signaling pathway (hsa04330). Particularly, pathways in cancer were regulated by most of the deregulated miRNAs in each MPRN, including 31 of 36 miRNAs shared by −β-catenin and −STAT3. In addition, four pathways were shared by −β-catenin and −CBP, including cell cycle (hsa04110), MAPK signaling pathway (hsa04010), p53 signaling pathway (hsa04115), and proximal tubule bicarbonate reclamation (hsa04964). Especially, miRNAs regulating p53 signaling pathway in both −β-catenin and −CBP were significantly in common (p = 1.371e–05; Fisher’s exact test). Moreover, genes deregulated by −β-catenin and −CBP in this pathway also showed overlap (p = 1.27e–05, Fisher’s exact test), including *SESN2*, *GADD45A*, *CDK4*, *SHISA5*, and *IGFBP3*. After investigating the distribution of genes deregulated by −β-catenin and –CBP within p53 signaling pathway (Fig. S6), we found that these two manipulations could affect many essential cellular processes such as cell cycle arrest, apoptosis, and DNA repair and damage prevention.

Finally, we calculated the number of miRNAs and pathways shared by paired combinations of all the six manipulations, and determined the statistical significance for each pair with Fisher exact test (Fig. S7). Five pair MPRNs showed significant overlap of functional miRNAs, while only MPRNs responding to −β-catenin and –CBP shared pathways significantly. These results implied that different interventions may activate the same set of miRNAs to regulate biological pathways. Although there were no pathways or miRNAs shared by all six MPRNs, three miRNAs and two pathways were shared by four MPRNs (Fig. S7). MiR-424, -130b, and -143 were common to −miR-23b, −β-catenin, −CBP, and −STAT3; miR-130b and -143 are known glioma risk factors [44], [51], and miR-424 has tumor-suppressor function [52]. The two pathways were cell cycle and protein processing in endoplasmic reticulum, with the former being shared by +miR-181d, −miR-23b, −β-catenin, and –CBP, and the latter being shared by +miR-181d, −miR-23b, −β-catenin, and –STAT3. Only one gene in cell cycle, *CDKN2D*, was differentially expressed in the same direction, being downregulated by all the four manipulations. Since the expression of *CDKN2D* is dependent on cell cycle phase, with expression lowest at mid-G1 and maximal during S-phase [53], the results implied that G1 arrest may be induced in glioma as a result of these four interventions. Another gene, *HSP90B1*, in protein processing in endoplasmic reticulum, was differentially expressed in the same direction, being downregulated, under miR-181d, −miR-23b, −β-catenin, and –STAT3. As a member of HSP90 heat shock protein family whose expression is upregulated in various cancers [54], the downregulation of *HSP90B1* revealed the consistency of these four manipulations on glioma suppression. As an example of functional crosstalk between miRNA- and TF-targeted manipulations, mRNAs with altered expression that were associated with protein processing in endoplasmic reticulum were examined (Figure 4C). Five miRNAs–miR-181c, -181d, -186, -30b and -30d–were found to regulate this pathway; except for miR-30b, all of these occurred in more than one condition.

### Response frequency and properties of MPRNs

**Figure 5:**
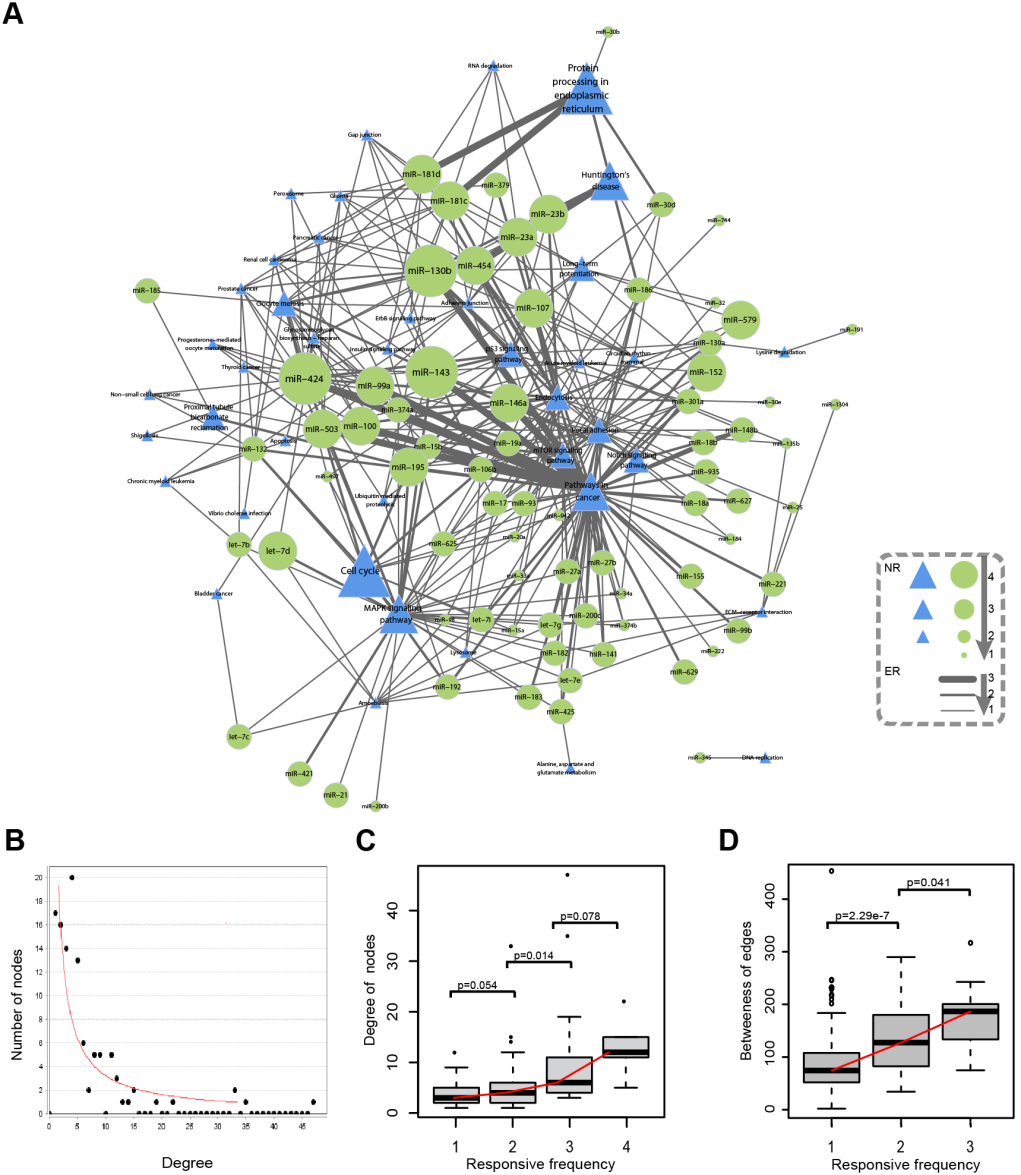
The comprehensive MPRN (cMPRN) and correlation between response frequency of miRNA-pathway regulations and their network centrality. (A) cMPRN was generated by integrating the six MPRNs corresponding to each experimental manipulation. Green circles and blue triangles represent functional miRNAs and target pathways, respectively. Node size and edge thickness are correlated with response frequency. (B) The degree distribution of cMPRN follows the power law. (C) The degree of nodes (including pathways and miRNAs) was positively correlated with their response frequency to different manipulations. (D) The betweenness of edges (miRNA-pathway regulation) was positively correlated with their response frequency.

A comprehensive MPRN (cMPRN) containing 41 pathways and 75 miRNAs was constructed by integrating MPRNs corresponding to all the six manipulations (Figure 5A). Firstly, we analyzed the topological characteristics of the cMPRN, including the distribution of node degree, shortest path length between two nodes, and edge betweenness. The distribution of node degree could be described by a power law equation with a slope of −0.975 and R^2^ = 0.753, indicating that cMPRN possessed the scale-free character of biological network (Figure 5B). On the other hand, biological network differs from random network in that the former has small-world feature, allowing the rapid spread of disturbances [55]. By preserving the number of direct neighbors for each miRNA and pathway in the original cMPRN using the edge-switching method, 1000 random networks were generated. We found that the average shortest path length of cMPRN was shorter than those of random networks (Fig. S8). Therefore, cMPRN was characterized with small-world feature of biological network as well [56].

All miRNAs and pathways in cMPRN were classified into four groups based on their response frequency (*i.e.*, number of occurrences) to different manipulations. We found that the number of miRNAs and pathways in each group was negatively correlated their response frequency (Fig. S9A). More importantly, a positive correlation between network degree and response frequency was found, indicating that nodes with higher response frequency were more likely to be hubs in cMPRN (Figure 5C). To investigate the respective contributions of miRNAs and pathways to this finding, the same analysis was applied independently to miRNAs and pathways. The results showed that the number of pathways in each group was more negatively correlated, while the degree distribution of miRNAs in each group was more positively correlated, with response frequency (Fig. S10). These results indicated that only a small number of pathways were influenced by multiple interventions; more frequently responsive pathways were not regulated by a large number of miRNAs, but rather by a select subset of them. MiRNAs were more likely than pathways to be simultaneously affected by different manipulations, and miRNAs regulating a larger number of pathways were more frequently affected by a given manipulation.

Accordingly, miRNA-pathway regulatory associations were divided into three groups based on their response frequency, and the number of associations in each group was also found to be negatively correlated with response frequency (Fig. S9B). Moreover, we calculated the betweenness centrality of regulatory associations in each group, and found that the median betweenness of each group significantly increased with response frequency (Figure 5D). These results suggested that miRNA-pathway regulatory associations with higher response frequency were more likely to be central in cMPRN, and may play important roles in determining the response to a particular intervention.

### The core module in cMPRN predicts glioma patient survival

**Figure 6:**
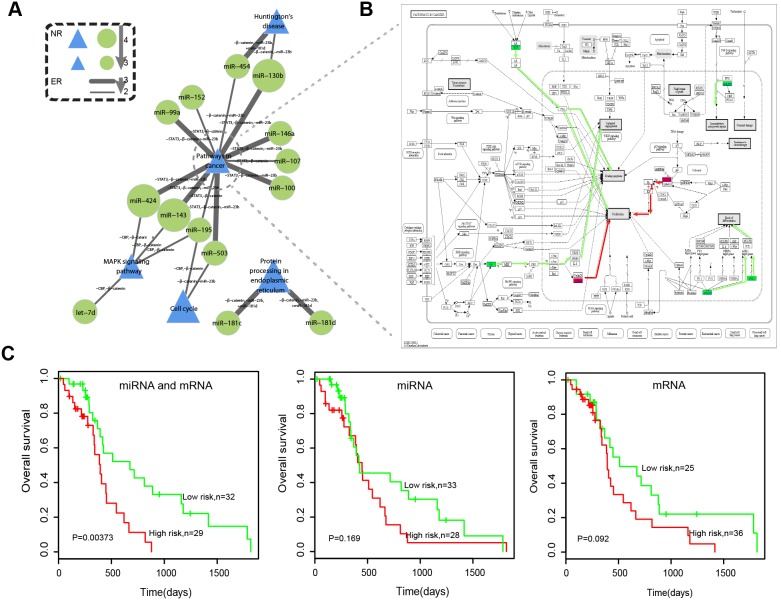
Association between the core module of cMPRN and glioma patient survival. (A) Green circles and blue triangles represent functional miRNAs and target pathways, respectively, in the core module. (B) Distribution of genes differentially expressed in the same direction under the three conditions sharing pathways in cancer: −STAT3, −β-catenin, and –miR-23b. Red and green rectangles represented genes that are up- and downregulated by the three experimental manipulations, while the white ones represent other genes within pathways in cancer. Red and green lines represent signal transduction downstream of these up- and downregulated genes, respectively. (C) Kaplan-Meier survival plot of the two subgroups of glioblastoma patients sorted by k-means clustering based on expression levels of either miRNA or mRNA signatures or both in the core module.

The clinical implications of specific miRNAs or pathways with high response frequency in cMPRN were evaluated. The network was filtered by retaining components with edge repeatability ≥2 and node repeatability ≥3 in all six manipulations, resulting in a core module of 21 miRNA-pathway regulatory associations, including 14 miRNAs and five pathways (Figure 6A). Of the 14 miRNAs, 11 were associated with glioma, *i.e.*, let-7d, and miR-100, -107, -130b, -143, -146a, -152, -181c, -181d, -195, and miR-99a. Although miR-454, -424, and -503 have not been implicated in gliomagenesis, they are involved in brain development, cancer, or other types of brain disease [57]–[59], and may therefore represent novel glioma-associated miRNAs. In addition, all five pathways in this core module were associated with glioma [60]–[63] as well as some regulatory associations, *e.g.*, the regulation of miR-195 to cell cycle [60]. Pathways in cancer (hsa05200) were shared by −STAT3, −β-catenin, and −miR-23b, and were regulated by the largest number of miRNAs, including seven common miRNAs shared by these three manipulations (miR-424, -143, -503, -99a, -130b, -146, and miR-100). In particular, four genes within pathways in cancer were differentially expressed in the same direction under all the three conditions, including *HSP90B1*, *ARAF*, and *RUNX1*, being downregulated, and *CDK4*, being upregulated under these three manipulations. After investigating the distribution of three downregulated genes within pathways in cancer, we found that *HSP90B1* was involved in promoting cell proliferation and evasion of apoptosis, *ARAF* (*RAF*) promoted sustained angiogenesis, and *RUNX1* induced differentiation and conferred tumor cells with an insensitivity to anti-growth signals (Figure 6B). Thus, the downregulation of these genes could suppress glioma by inhibiting cell proliferation and angiogenesis, inducing apoptosis, and weakening the block of differentiation, making glioma cells more sensitive to anti-growth signals, and are thus potential therapeutic targets. *ARAF* encodes a scaffold that stabilizes BRAF: CRAF heterodimers and regulates RAF signaling; and specific inhibitors have been successful in phase I/II clinical trials in cancer patients [64]. *RUNX1* is a major regulator of the glioma mesenchymal subtype [65], and was found to interact with STAT3 in the miRNA-mediated RNA-RNA interaction network [36]; it is therefore not unexpected that *RUNX1* expression was downregulated by blocking the activity of STAT3. The upregulated gene, *CDK4*, acts downstream of Raf and regulates cell proliferation (Figure 6B); thus, increased *CDK4* expression may negate the inhibition of cell proliferation resulting from downregulation of *ARAF*, leading instead to the survival of glioma cells. Previous studies have found that anti-cancer therapeutic approaches can have antagonistic effects on tumor malignancy, reducing primary tumor growth and simultaneously increasing invasiveness [66]. Thus, the opposite effect of three downregulated genes and the upregulated one on tumor malignancy within pathways in cancer may provide more inspiration for the development of anti-cancer therapeutics.

The core module identified in cMPRN was comprised of critical miRNA-pathway regulations activated by multiple manipulations, and therefore may play an important role in glioma. Hence, we evaluated the ability of this module to predict glioma patient prognosis. An independent dataset of 61 GBM patients that have received adjuvant therapy was downloaded from TCGA [33], which contained information on survival time and paired expression profiles of miRNAs and mRNAs. Firstly, we investigated the correlation of mRNA and miRNA expressions in each experimental sample to that of the glioblastoma patients for survival analysis, and tested the significance by shuffling the expression of mRNAs and miRNAs in each experimental sample 1000 times. The result revealed that both the expression of mRNA and miRNA are positively correlated to that of the glioblastoma patients (Fig. S11). All the miRNAs in the core module were assigned as its miRNA signatures. For each miRNA-pathway regulation in the core module, genes within the pathway that were deregulated in the same direction under manipulations sharing the regulation were identified as mRNA signatures. A total of 14 miRNA signatures and 26 mRNA signatures were obtained from this core module (Table S1). The 61 GBM patients were assigned to two subgroups by k-means clustering based on the expression levels of miRNA and mRNA signatures. Kaplan-Meier method and log-rank test were used to estimate the difference in survival time between the two subgroups (Figure 6C, left). The p value of 0.00373 was highly significant, and indicated that miRNA and mRNA signatures in this core module have strong potential for distinguishing glioma patients with good and poor prognosis. Moreover, we tested the independent prognostic values of miRNA and mRNA signatures, and found that each one alone had no predictive value (Figure 4C, middle and right). The results indicated that the core miRNA-pathway regulation module may play important roles in glioma therapeutics.

Furthermore, we assessed the prognostic association between the module signature and other known clinical risk factors, including age and karnofsky performance score (KPS), for glioblastoma progression with univariate and multivariate analyses. The results of multivariate analysis revealed that the module signature remained an independent prognostic risk factor for glioblastoma patient survival (P = 1.12e-6; Table S2), although age and KPS were also significantly associated with the survival of glioblastoma patients in univariate analysis.

It has been reported that RNAs are always co-regulated by miRNAs in cellular context, and thus compose competing endogenous RNAs (ceRNAs) system [36]. To investigate the influence of ceRNA system on our experiment, we downloaded the microRNA-mediated network of RNA-RNA interactions identified from sample-matched gene and miRNA expression profiles of glioblastoma [36], and estimated the proportion of mRNAs in each MPRN as well as mRNA signatures of the core module related to the co-regulation of functional miRNAs. The results revealed that mRNA signatures in the core module were more related to the RNA-RNA interactions mediated by miRNA signatures (Fig. S12), suggesting that mRNA and miRNA signatures in the core module could be important players in the ceRNA system.

## Discussion

In our study, six targeted manipulations were carried out based on U87 cell lines, which are widely used cell lines in previous researches of glioma [67], [68]. We observed that most of the miRNAs and pathways in each MPRN have been reported to be associated with glioma (Figure 3D), indicating that our results are in accordance with previous studies. In addition, we have downloaded independent miRNA and mRNA profiles of glioblastoma patient from TCGA, and demonstrated that the expression of module signatures identified in our study based on U87 cell lines were significantly associated with the prognosis of glioblastoma patients (Figure 6C), implying that our results could facilitate the identification of useful biomarkers for glioma therapy. Moreover, we found that the expression of mRNAs and miRNAs in each experimental sample were positively correlated to that of the glioblastoma patients (Fig. S11), indicating that the identified MPRNs may have implications regarding the identification of relevant targets for glioma therapy.

Extensive functional synergy and crosstalk was observed between these MPRNs, and the shared miRNA-pathway regulatory associations could provide new perspectives for estimating the functional coordination between different therapeutic interventions [69]. For example, β-catenin is the main effector of Wnt signaling pathway, and could initiate the transcription of downstream genes by binding to members of T-cell factor (Tcf)/lymphoid enhancement factor family, which could be facilitated by the acetylation of adjacent histones mediated by CBP. ICG001 treatment could lead to the downregulation of β-catenin/Tcf signaling by binding to CBP, while FH535 could suppress β-catenin activity directly. Therefore, the significant overlap between MPRNs activated by these two manipulations was in accordance with their functional synergism (Figs. 2B, S5).

The results provide a basis for identifying functional associations between different miRNAs. For example, miR-17 and -106b were both downregulated and acted cooperatively to regulate focal adhesion (hsa04510) and NOD-like receptor signaling pathways (hsa04621) by mediating MAPK9 expression under the manipulation of miR-181d overexpression(Fig. S13). It has been suggested that miR-17 and -106b were generated from two homologous miRNA clusters (miR-17-92 and miR-106b-25, respectively), and they can coordinately promote G1/S transition by suppressing the expression of MAPK9 [70], [71]. Moreover, their downregulation has been shown to suppress growth in human glioma cells [72], [73]. Therefore, downregulation of miR-17 and -106b may synergistically inhibit glioma malignancy after over expressing miR-181d.

These findings can also be used to improve the efficacy evaluation of specific interventions on glioma therapy. Currently, measuring changes in expression of direct target genes after a manipulation is a standard method of validating its effect [8], [60], [74]; however, the construction of MPRNs can allow indirect effects, as well as functional synergism and cross-regulatory effects, to be examined. For example, as a tumor suppressor miR-181d was significantly upregulated after knocking down miR-23b, which may synergize with miR-23b suppression to inhibit the growth of glioma cells. In contrast, expression levels of miR-181 family members, including miR-181d, were significantly reduced after blocking the activity of β-catenin, suggesting that miR-181d might antagonize the effects of β-catenin inhibition, thereby inducing drug resistance. The present work could help to identify molecules that facilitate or oppose the activity of specific pathway, as well [75]. For example, the activity of MAPK signaling pathway was inclined to be inhibited after knocking down miR-21, but tended to be activated after blocking the activity of β-catenin or CBP, indicating that these interventions can have either synergistic or antagonistic effects on this pathway (Fig. S3).

Since some popular cancer drugs with validated anti-tumorigenic effects have been shown to elicit pro-tumorigenic phenotypes in specific glioma cells [76], an examination of the functional MPRNs responding to a given pharmacological manipulation can lead to a better understanding of its multiple effects, thus provide inspirations for the development of multi-drug treatment strategies, as well as the identification of biomarkers that can accurately predict prognosis in glioma patients. It has been observed that the responses of miRNAs (after 24–48 h) were delayed with respect to mRNAs (12–24 h) after targeted molecular intervention, and that both the expression of miRNAs and mRNAs were not altered significantly after 72 h [77]. Since we only investigated the expression of mRNA and miRNA at 72 h after each targeted intervention, our present work could not be utilized to analyze the gradual change of mRNA and miRNA expression after targeted molecular intervention. Moreover, our work mainly focused on the experiments with validated effect on glioma inhibition, further experiments with contrary molecular intervention such as transfecting antagmirs to miR-181d into cells can help us to understand better on miRNAs’ regulatory role in gliomagenesis related to each targeted molecule.

## Supporting Information

Figure S1 **The expression changes of three targeted miRNAs, miR-181d,** -**21, and** -**23b, under each manipulation.**
(TIF)

Figure S2 **Global changes in the expression of miRNAs and mRNAs after each experimental manipulation.** The distribution of (A) miRNA and (B) mRNA expressions in each experimental sample was shown in boxplot. No significant differences were observed in miRNA or mRNA expressions between paired case-control samples (p>0.1; Wilcoxon test). The log2 fold changes of (C) miRNAs and (D) mRNAs after each manipulation were shown in density plot.
(TIF)

Figure S3 **Distribution of differentially expressed genes in MAPK signaling pathway.** The distribution of genes deregulated by inhibition of (A) miR-21, (B) CREB-binding protein (CBP), and (C) β-catenin are shown. Red, green and white rectangles represent up-, down-regulated and other genes in the pathway, respectively. (D) Overlap between genes that are up- and downregulated by inhibition of CBP and β-catenin within MAPK signaling pathway.
(TIF)

Figure S4 **Distribution of differentially expressed genes whithin pathways in cancer after inhibiting the activity of signal transducer and activator of transcription 3 (STAT3).** Red, green and white rectangles represent up-, down-regulated genes and other genes within this pathway, respectively.
(TIF)

Figure S5 **The significance of functional miRNA-pathway regulations from each MPRN in 1000 randomizations.**
(TIF)

Figure S6 **The overlap of differentially expressed genes between manipulations of inhibiting the activities of β-catenin (–β-catenin) and CREB-binding protein (–CBP) within p53 signaling pathway.** Red rectangles represent genes deregulated by both manipulations, while the yellow and purple ones respectively represent genes deregulated by –β-catenin or –CBP alone. The white rectangles represent other genes within this pathway.
(TIF)

Figure S7 **Summary of the functional synergy between MPRNs activated by each experimental manipulation.** According to the number of shared manipulations (Num), all the paired combinations were divided into four groups. Fisher’s exact test was used to calculate the statistical significance of the overlap between networks resulting from each pair of manipulations. *p<0.05.
(TIF)

Figure S8 **Average shortest path length of the comprehensive MPRN.**
(TIF)

Figure S9 **The number of (A) nodes and (B) edges with different response frequency in cMPRN.** The nodes included functional miRNAs and target pathways. The edges were functional miRNA-pathway regulations.
(TIF)

Figure S10 **Contributions of miRNAs and pathways to the positive correlation between response frequency and network centrality.** The degree distribution of (A) pathways and (B) miRNAs in cMPRN. The number of (C) pathways and (D) miRNAs with different response frequency to targeted manipulations in cMPRN. The median degree of each group of (E) pathways and (F) miRNAs with different response frequency.
(TIF)

Figure S11 **The average correlation of mRNA/miRNA expression in all the eight samples to that of glioblastoma patients.** The histogram of average expression correlation of mRNA (A) and miRNA (B) in all the eight samples to that of glioblastoma patients in 1000 randomization. The red arrow represents the real average expression correlation of (A) mRNAs and (B) miRNAs in all the eight samples to that of glioblastoma patients.
(TIF)

Figure S12 **The proportion of differentially expressed mRNAs in each MPRN related to ceRNA network mediated by functional miRNAs.**
(TIF)

Figure S13 **The regulation of miR-17 and miR-106b to MAPK9 and their effect on biological pathways.** Blue triangles, green circles, and green and red hexagons represent pathways and downregulated miRNAs, and down- and upregulated genes, respectively.
(TIF)

Table S1 **MiRNA and mRNA signatures of each miRNA-pathway regulation in the core module of the MPRN.**
(XLS)

Table S2 **Cox regression analyses of module signature, age, and karnofsky performance score (KPS) in the glioblastoma patient cohort.**
(XLS)
