# Supplementary figures and images for: Identification of a Core miRNA-Pathway Regulatory Network in Glioma by Therapeutically Targeting miR-181d, miR-21, miR-23b, β-Catenin, CBP, and STAT3
Source: PLoS One. 2014 Jul 9;9(7):e101903. doi: 10.1371/journal.pone.0101903 (PMC4090169; doi:10.1371/journal.pone.0101903)

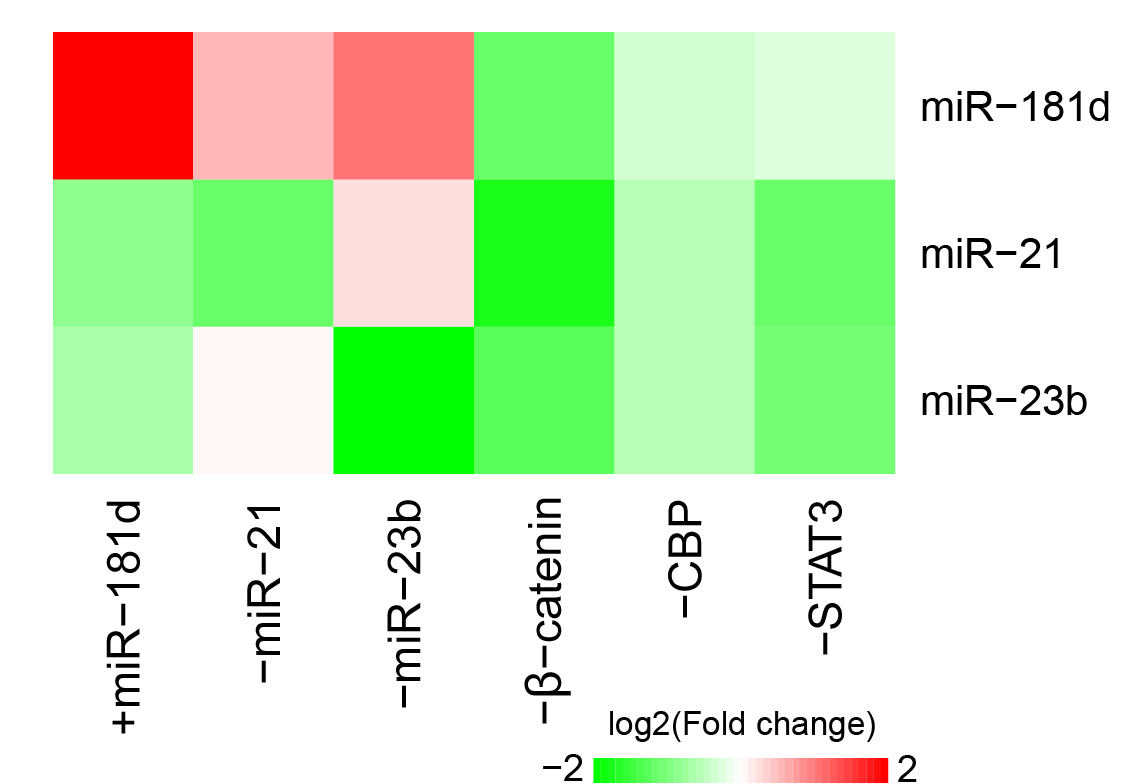

Supplement: Figure S1 — The expression changes of three targeted miRNAs, miR-181d, -21, and -23b, under each manipulation. (TIF) [file pone.0101903.s001.tif]

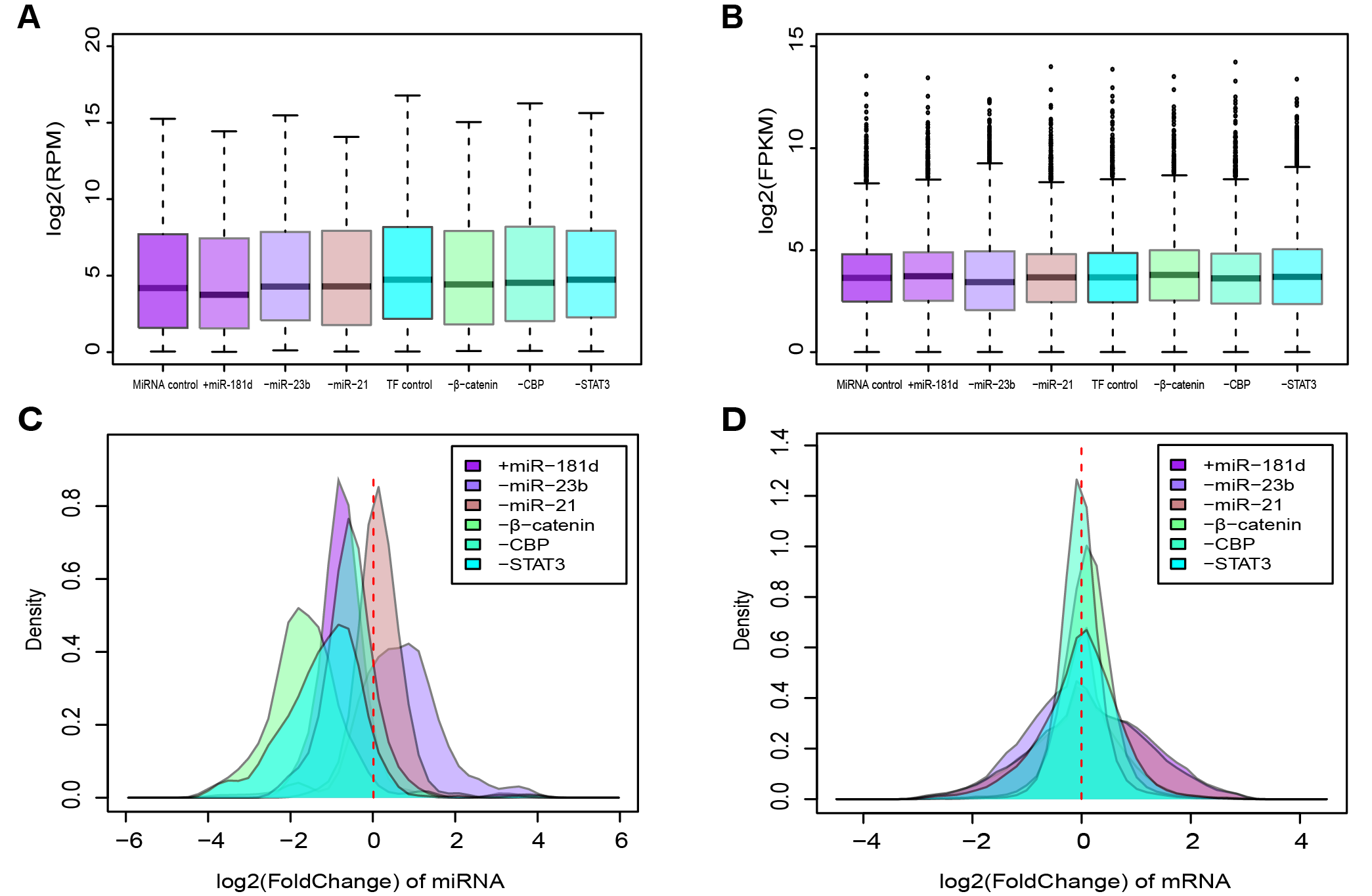

Supplement: Figure S2 — Global changes in the expression of miRNAs and mRNAs after each experimental manipulation. The distribution of (A) miRNA and (B) mRNA expressions in each experimental sample was shown in boxplot. No significant differences were observed in miRNA or mRNA expressions between paired case-control samples (p>0.1; Wilcoxon test). The log2 fold changes of (C) miRNAs and (D) mRNAs after each manipulation were shown in density plot. (TIF) [file pone.0101903.s002.tif]

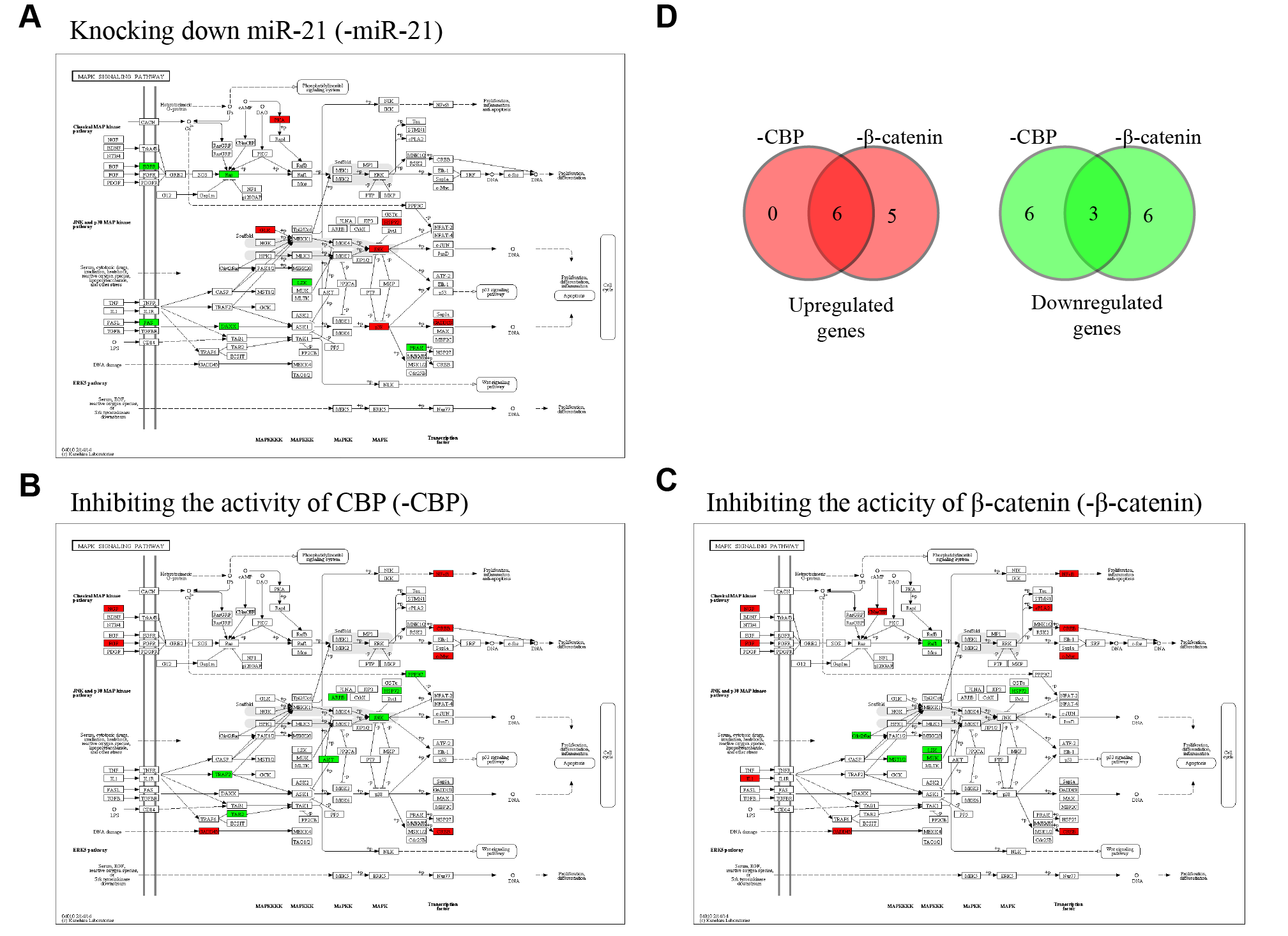

Supplement: Figure S3 — Distribution of differentially expressed genes in MAPK signaling pathway. The distribution of genes deregulated by inhibition of (A) miR-21, (B) CREB-binding protein (CBP), and (C) β-catenin are shown. Red, green and white rectangles represent up-, down-regulated and other genes in the pathway, respectively. (D) Overlap between genes that are up- and downregulated by inhibition of CBP and β-catenin within MAPK signaling pathway. (TIF) [file pone.0101903.s003.tif]

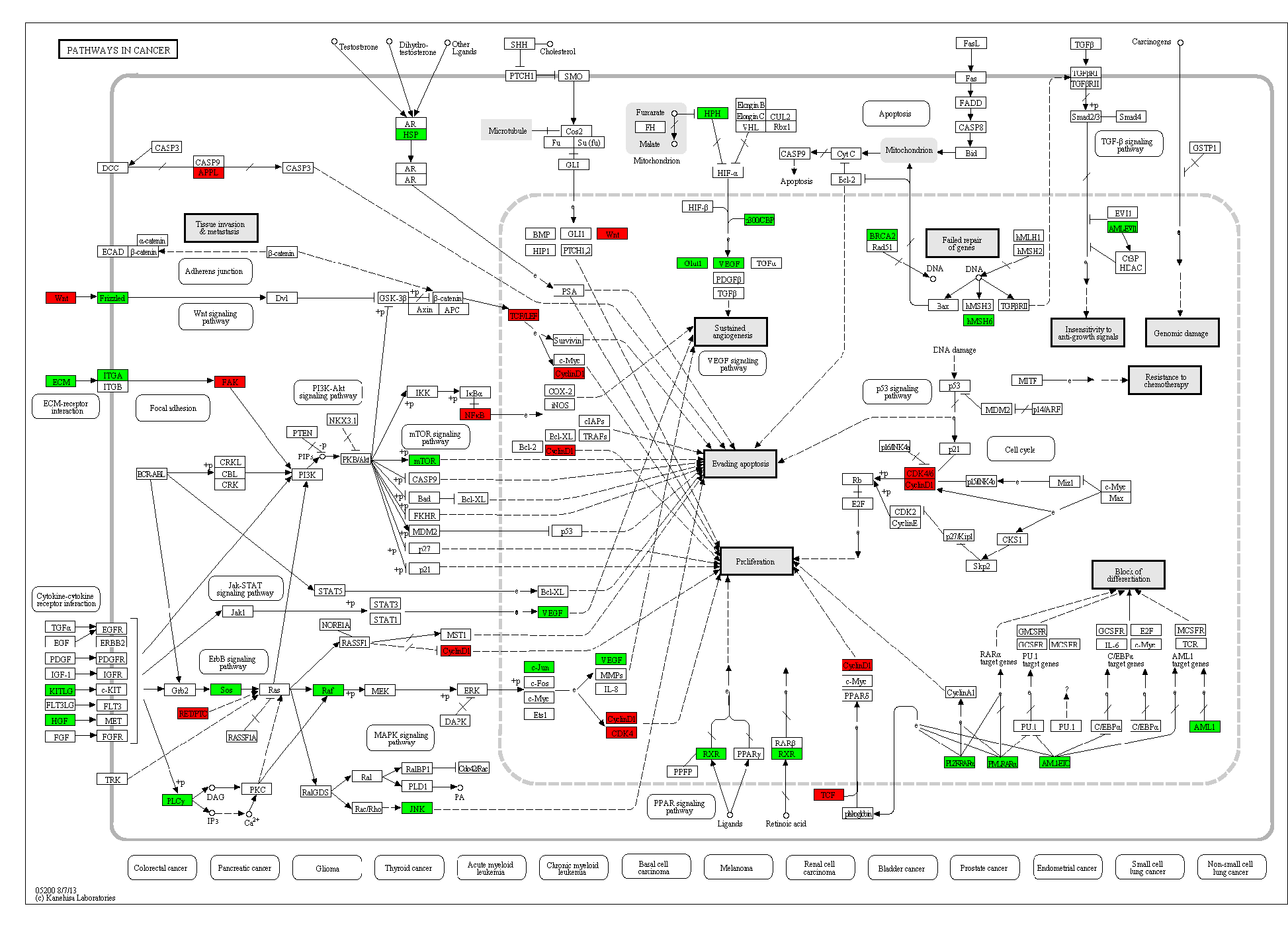

Supplement: Figure S4 — Distribution of differentially expressed genes whithin pathways in cancer after inhibiting the activity of signal transducer and activator of transcription 3 (STAT3). Red, green and white rectangles represent up-, down-regulated genes and other genes within this pathway, respectively. (TIF) [file pone.0101903.s004.tif]

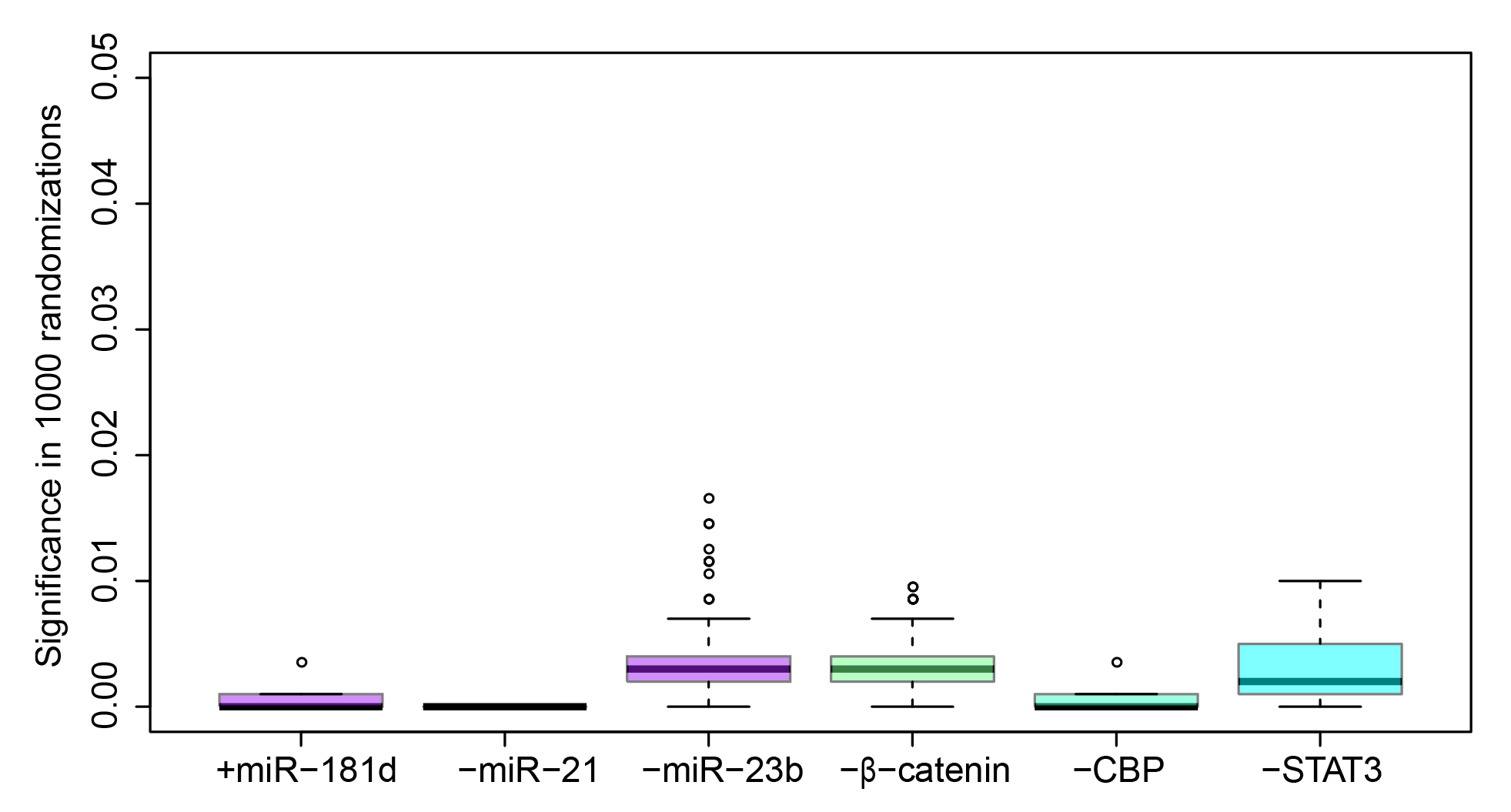

Supplement: Figure S5 — The significance of functional miRNA-pathway regulations from each MPRN in 1000 randomizations. (TIF) [file pone.0101903.s005.tif]

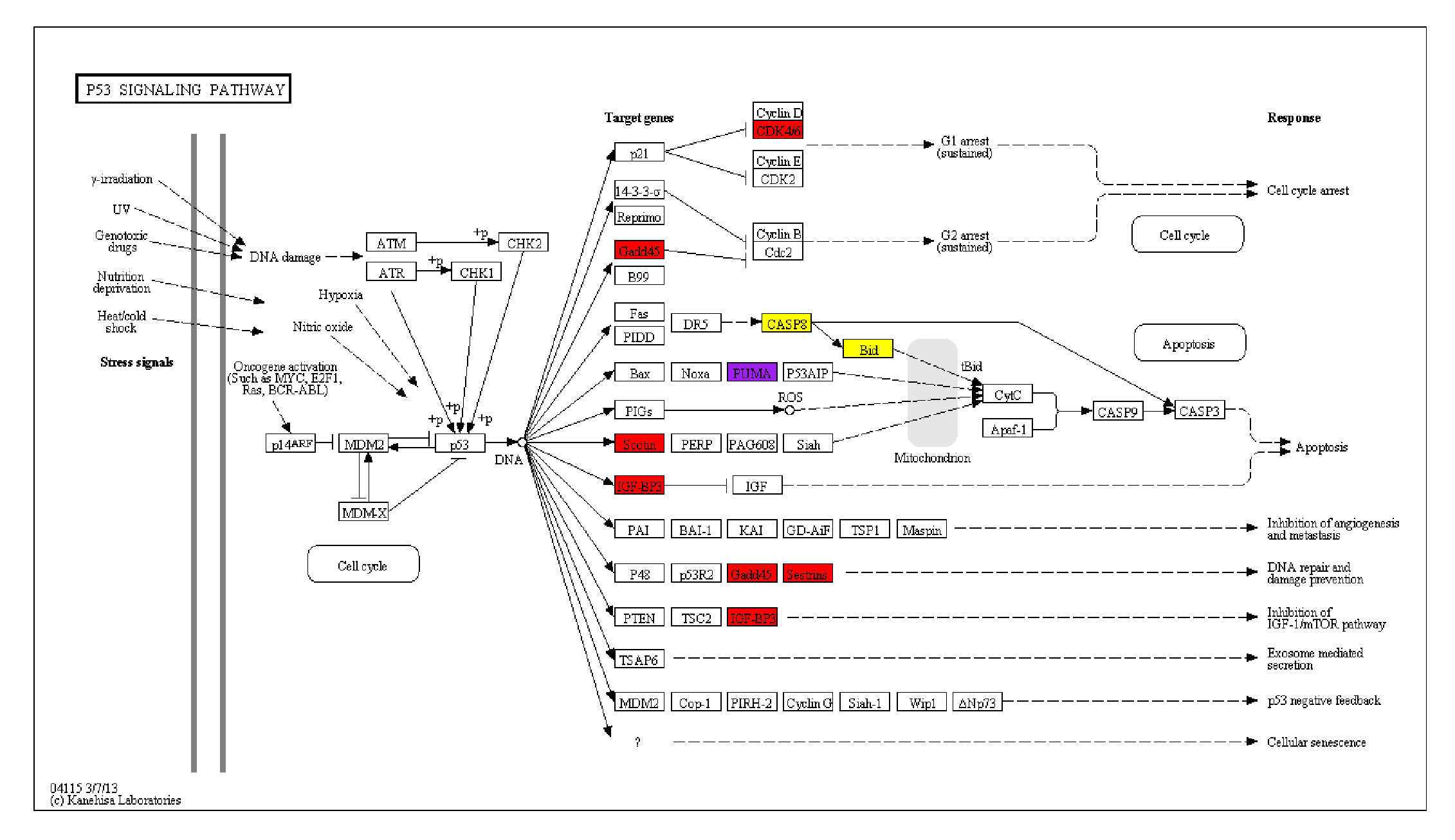

Supplement: Figure S6 — The overlap of differentially expressed genes between manipulations of inhibiting the activities of β-catenin (–β-catenin) and CREB-binding protein (–CBP) within p53 signaling pathway. Red rectangles represent genes deregulated by both manipulations, while the yellow and purple ones respectively represent genes deregulated by –β-catenin or –CBP alone. The white rectangles represent other genes within this pathway. (TIF) [file pone.0101903.s006.tif]

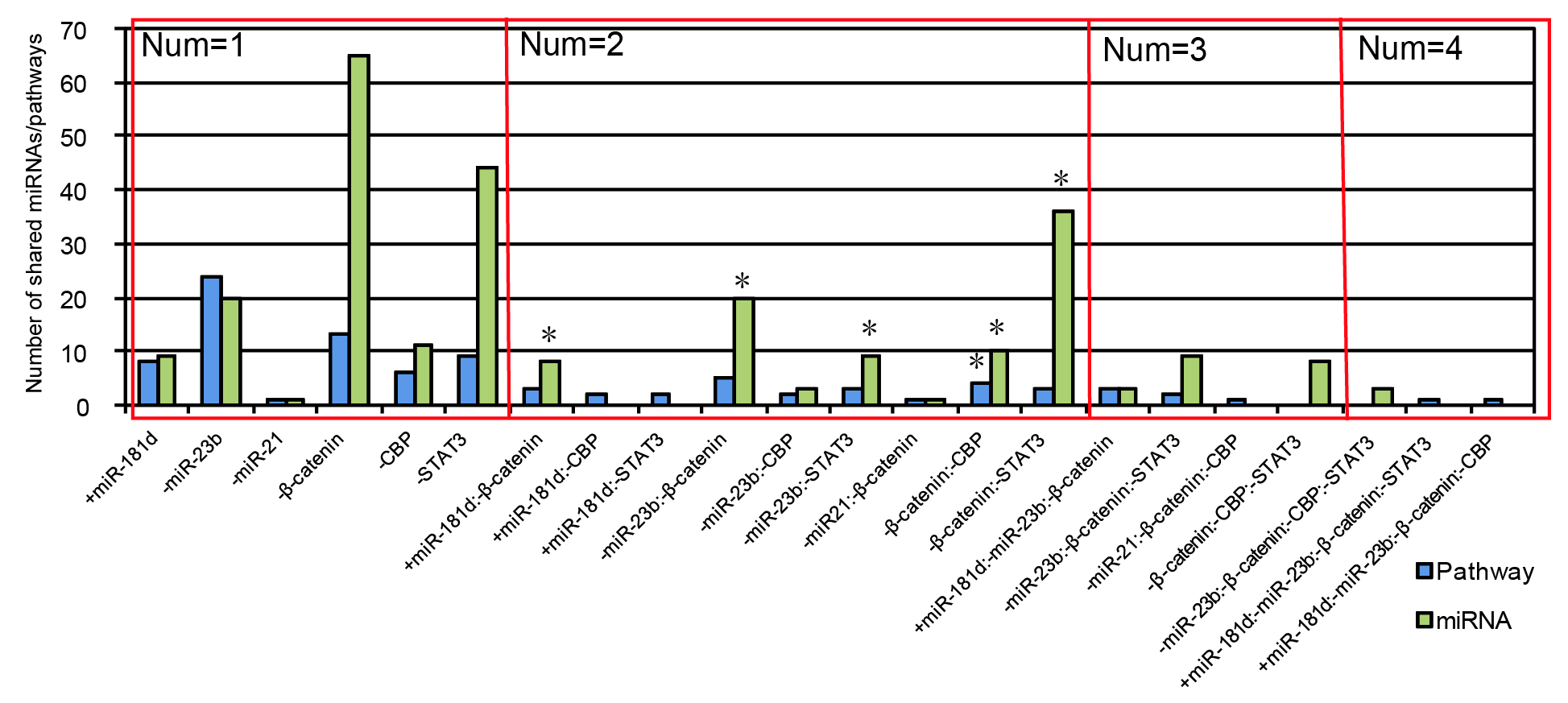

Supplement: Figure S7 — Summary of the functional synergy between MPRNs activated by each experimental manipulation. According to the number of shared manipulations (Num), all the paired combinations were divided into four groups. Fisher’s exact test was used to calculate the statistical significance of the overlap between networks resulting from each pair of manipulations. *p<0.05. (TIF) [file pone.0101903.s007.tif]

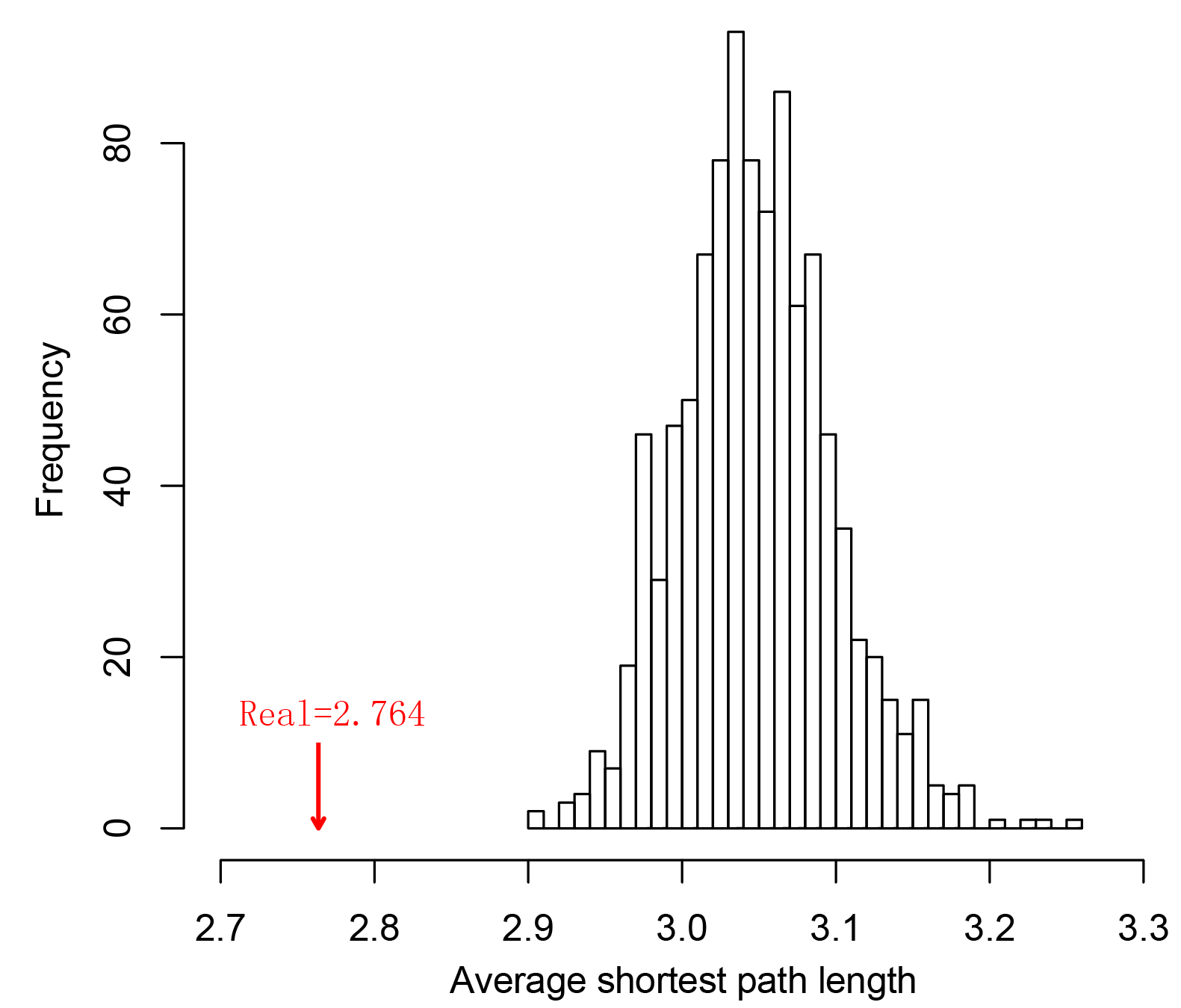

Supplement: Figure S8 — Average shortest path length of the comprehensive MPRN. (TIF) [file pone.0101903.s008.tif]

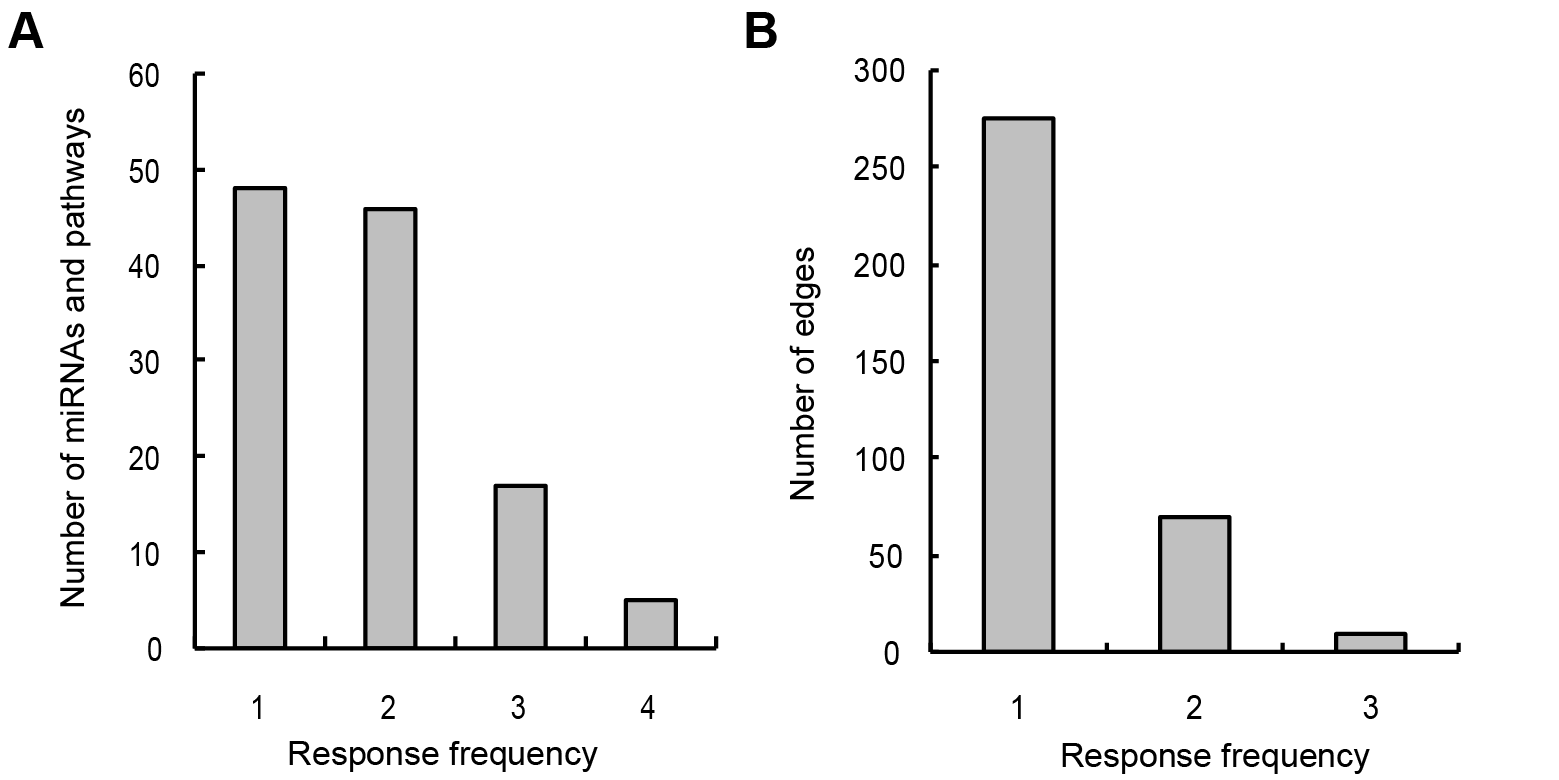

Supplement: Figure S9 — The number of (A) nodes and (B) edges with different response frequency in cMPRN. The nodes included functional miRNAs and target pathways. The edges were functional miRNA-pathway regulations. (TIF) [file pone.0101903.s009.tif]

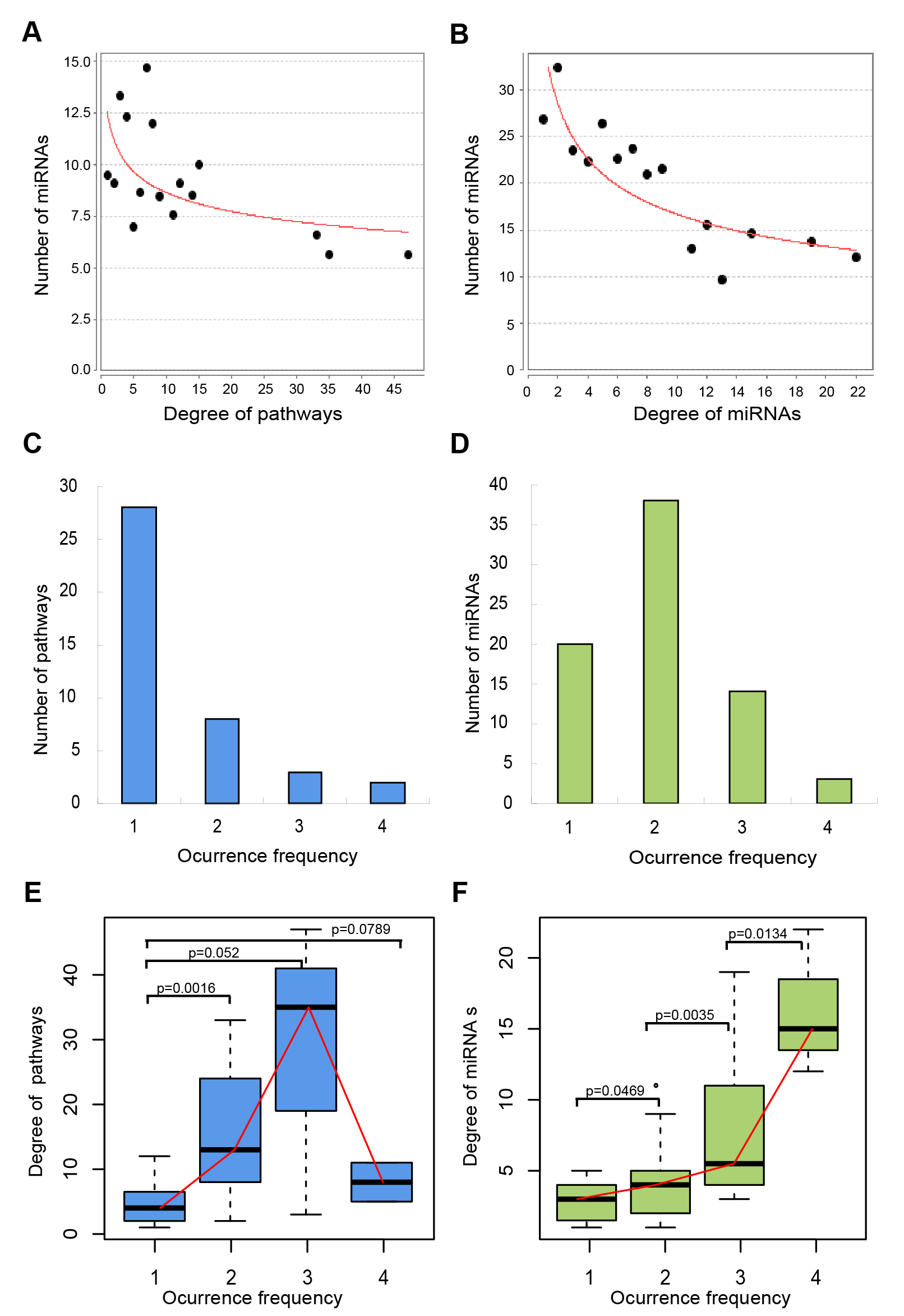

Supplement: Figure S10 — Contributions of miRNAs and pathways to the positive correlation between response frequency and network centrality. The degree distribution of (A) pathways and (B) miRNAs in cMPRN. The number of (C) pathways and (D) miRNAs with different response frequency to targeted manipulations in cMPRN. The median degree of each group of (E) pathways and (F) miRNAs with different response frequency. (TIF) [file pone.0101903.s010.tif]

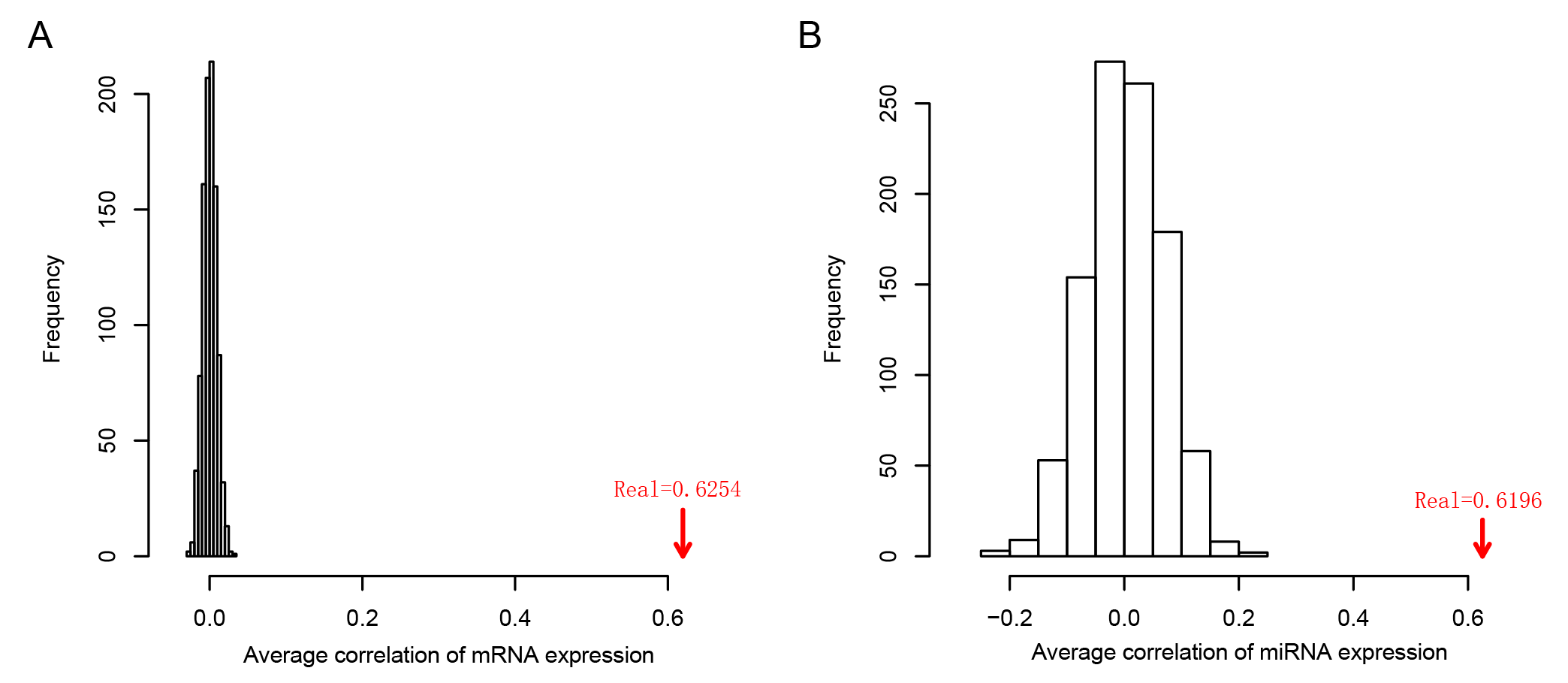

Supplement: Figure S11 — The average correlation of mRNA/miRNA expression in all the eight samples to that of glioblastoma patients. The histogram of average expression correlation of mRNA (A) and miRNA (B) in all the eight samples to that of glioblastoma patients in 1000 randomization. The red arrow represents the real average expression correlation of (A) mRNAs and (B) miRNAs in all the eight samples to that of glioblastoma patients. (TIF) [file pone.0101903.s011.tif]

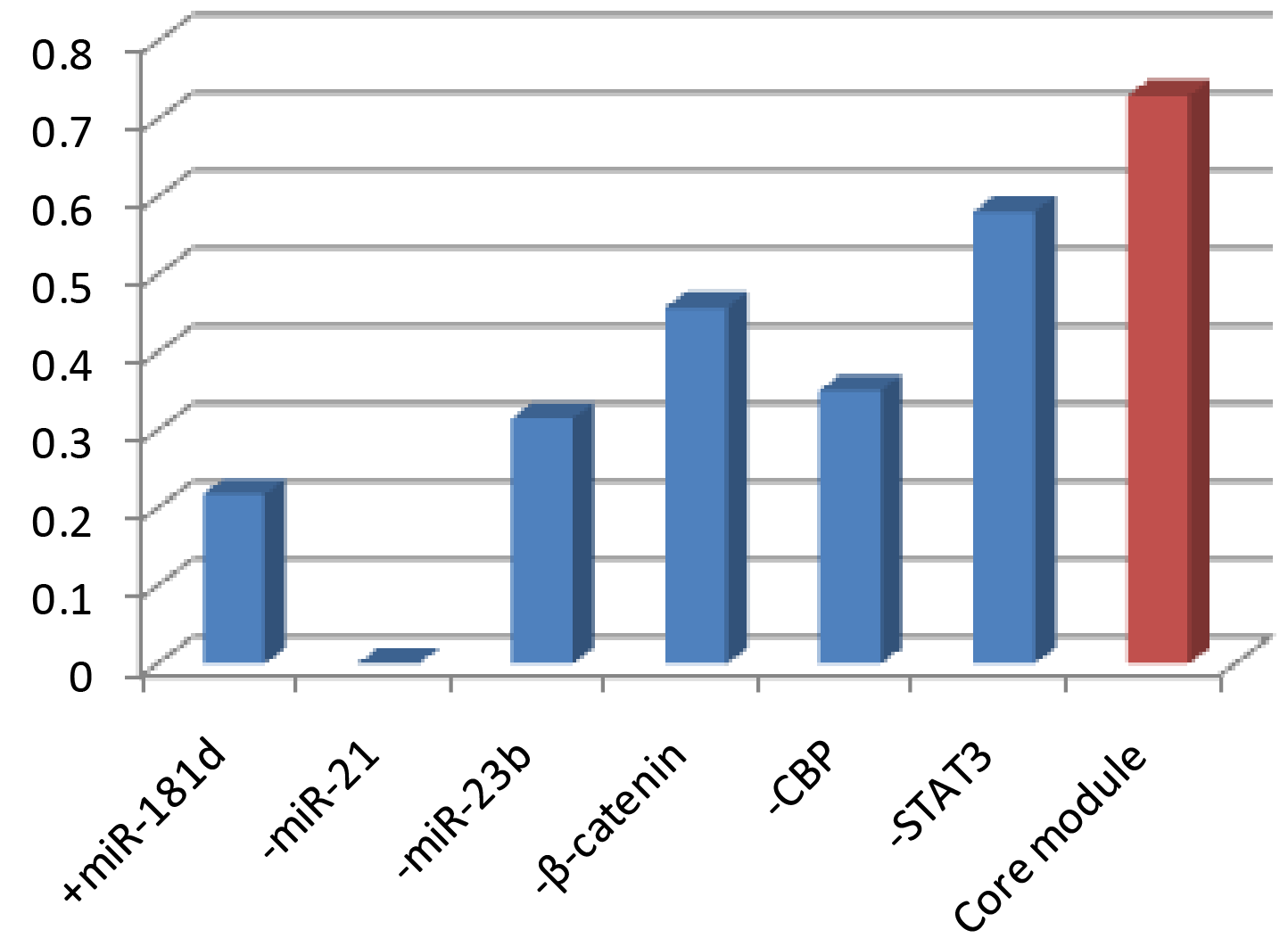

Supplement: Figure S12 — The proportion of differentially expressed mRNAs in each MPRN related to ceRNA network mediated by functional miRNAs. (TIF) [file pone.0101903.s012.tif]

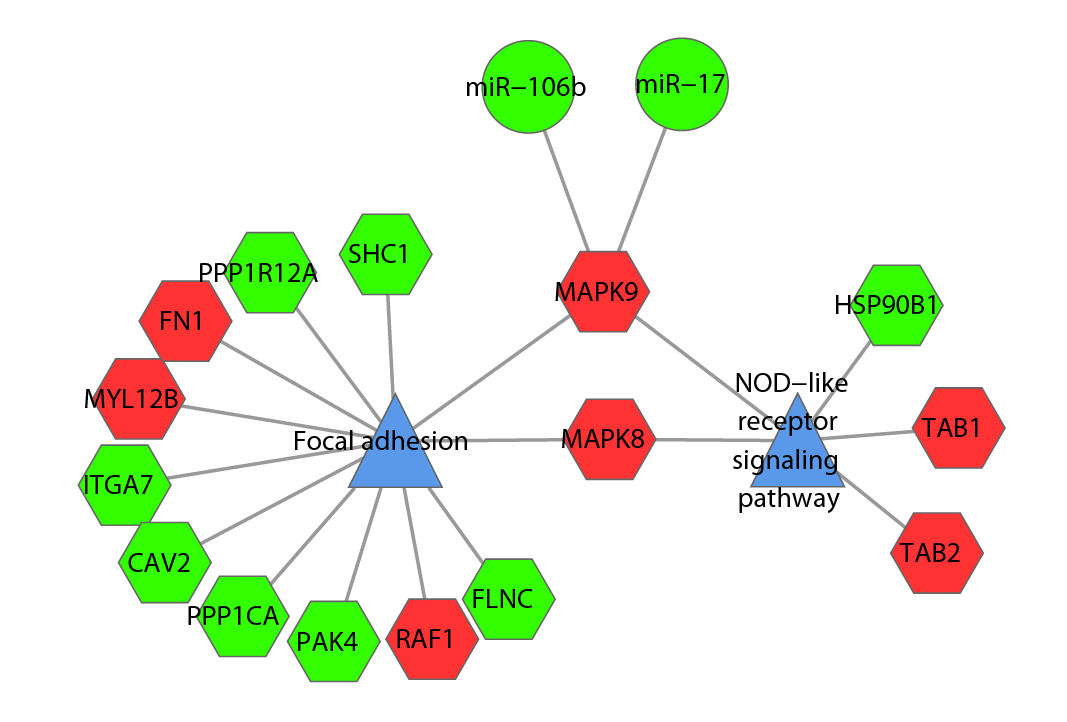

Supplement: Figure S13 — The regulation of miR-17 and miR-106b to MAPK9 and their effect on biological pathways. Blue triangles, green circles, and green and red hexagons represent pathways and downregulated miRNAs, and down- and upregulated genes, respectively. (TIF) [file pone.0101903.s013.tif]
